# Early ERPs to faces: aging, luminance, and individual differences

**DOI:** 10.3389/fpsyg.2013.00268

**Published:** 2013-05-14

**Authors:** Magdalena M. Bieniek, Luisa S. Frei, Guillaume A. Rousselet

**Affiliations:** ^1^Institute of Neuroscience and Psychology, College of Medical, Veterinary and Life Sciences, University of GlasgowGlasgow, UK; ^2^Institute of Health and Wellbeing, College of Medical, Veterinary and Life Sciences, University of GlasgowGlasgow, UK

**Keywords:** event related potentials, aging, luminance, pupil size, senile miosis, retinal illuminance, individual differences, N170

## Abstract

Recently, Rousselet et al. reported a 1 ms/year delay in visual processing speed in a sample of healthy aged 62 subjects (Frontiers in Psychology 2010, 1:19). Here, we replicate this finding in an independent sample of 59 subjects and investigate the contribution of optical factors (pupil size and luminance) to the age-related slowdown and to individual differences in visual processing speed. We conducted two experiments. In experiment 1 we recorded EEG from subjects aged 18–79. Subjects viewed images of faces and phase scrambled noise textures under nine luminance conditions, ranging from 0.59 to 60.8 cd/m^2^. We manipulated luminance using neutral density filters. In experiment 2, 10 young subjects (age < 35) viewed similar stimuli through pinholes ranging from 1 to 5 mm. In both experiments, subjects were tested twice. We found a 1 ms/year slowdown in visual processing that was independent of luminance. Aging effects became visible around 125 ms post-stimulus and did not affect the onsets of the face-texture ERP differences. Furthermore, luminance modulated the entire ERP time-course from 60 to 500 ms. Luminance effects peaked in the N170 time window and were independent of age. Importantly, senile miosis and individual differences in pupil size did not account for aging differences and inter-subject variability in processing speed. The pinhole manipulation also failed to match the ERPs of old subjects to those of young subjects. Overall, our results strongly suggest that early ERPs to faces (<200 ms) are delayed by aging and that these delays are of cortical, rather than optical origin. Our results also demonstrate that even late ERPs to faces are modulated by low-level factors.

## Introduction

One of the most prominent phenomena associated with aging is a progressive cognitive slowing. Age-related slowing is visible across a variety of cognitive tasks (Salthouse and Ferrer-Caja, 2003), and seems to be linked to a number of neurodegenerative changes, including total brain volume shrinkage (Resnick et al., 2003), and alterations in gray matter (Brickman et al., 2008; Chee et al., 2009) and white matter integrity (Davis et al., 2009; Peters, 2009; Piguet et al., 2009; Salat et al., 2009). Whereas some brain regions, in particular pre-frontal areas, seem to suffer substantial age-related structural and functional deterioration, no significant shrinkage of primary visual cortex has been observed (Resnick et al., 2003; Raz et al., 2005, 2010). However, important neural changes in visual areas have been documented in animal models, including delayed latencies (Wang et al., 2005, 2006) and degradation in response selectivity of neurons in striate and extrastriate cortical areas (Schmolesky et al., 2000; Hua et al., 2006; Yu et al., 2006; Peters, 2009). In keeping with findings in animals, fMRI studies in humans have described weaker differentiation between categorical responses in old subjects (Park et al., 2004; Voss et al., 2008). Such changes in tuning may lead to longer processing times, following a model of perceptual decision by accumulation of evidence in neuronal populations (Perrett and Ashbridge, 1998). There is also more direct evidence from human ERP studies, showing delayed evoked responses to checkerboards (Sokol et al., 1981; Tobimatsu et al., 1993). These neural changes suggest an overall slowdown of perception with age (Rousselet et al., 2009, 2010), which in turn could affect higher cognitive functions, such as working memory (Gazzaley et al., 2008).

We can identify at least five main questions about age-related slowing of perception:

1. When and where in the brain does it start to manifest itself?
2. By how much do we slow down?
3. From what age do we slow down?
4. Why do we slow down?
5. What are the consequences?

In this study, we tried to address, to some extent, the first four questions by measuring event-related potentials (ERPs) to pictures of faces and noise textures. Previously, using a similar approach, Rousselet et al. (2009, 2010) reported age-related visual processing slowing of about 1 ms/year from age 20 onward. Aging effects started at about 120 ms post-stimulus onset, suggesting a cortical origin, and the potential involvement of face and object processing cortical areas. However, these results remain controversial. Several other studies did not find aging effects on the latencies of early ERPs to complex objects, such as faces (Chaby et al., 2001, 2003; Pfutze et al., 2002; Gao et al., 2009; Daniel and Bentin, 2010; Wiese et al., 2012). These studies observed age-related delays only at later stages of visual processing (>200 ms). Contrary to these negative results, several studies reported age-related latency increases of early ERPs to faces (Nakamura et al., 2001; Gazzaley et al., 2008; Wiese et al., 2008), letters (Falkenstein et al., 2006; Kolev et al., 2006), letter-number pairs (De Sanctis et al., 2008) and diffuse light flashes (Diaz and Amenedo, 1998).

Given the discrepancies in the literature, the first goal of our study was to replicate the age-related delays described in Rousselet et al. (2009, 2010) using an independent sample of subjects. The second goal of our study was to determine the origin of the aging effects, as well as the origin of the very large inter-individual differences we observed within age groups. There are many potential and non-mutually exclusive contributors to these effects, from bottom-up optical and neural factors, to various high-level explanations—see discussions in Rousselet et al. (2009, 2010). One particularly important factor is senile miosis, the age-related reduction in pupil size (Winn et al., 1994). Pupil size also varies considerably within the same age group (Winn et al., 1994). Hence, because senile miosis reduces retinal illuminance, it could contribute to the delays and the considerable within age-group individual differences in cortical processing speed found in previous studies. Several elements support this idea. First, delayed latencies of the early evoked potentials (<200 ms) have been observed in subjects with smaller pupils (Hawkes and Stow, 1981) and when subjects viewed stimuli through a pinhole aperture of 1.5 mm size (Vanmaele et al., 2003). Second, a link between luminance and the latencies of neuronal responses have been documented in animals using multi-focal ERG (Raz et al., 2002) and in humans using pattern ERG and ERP recordings (Froelich and Kaufman, 1991). Previous research has shown that decreasing luminance increases the latencies of neuronal responses in various cortical areas including V1 (Geisler et al., 2007), the superior colliculus (Marino et al., 2012) and the LIP—lateral intraparietal area (Tanaka et al., 2013). Importantly, it seems that age-related delays in retinal and cortical activity can be abolished after equating retinal illuminance between groups by using neutral density filters (Trick et al., 1986). In the same vein, Shaw and Cant (1980) reported that the age-related P100 delays observed at lower (5 cd/m^2^) luminance levels were considerably reduced at higher luminance (90 cd/m^2^). However, this finding was challenged by a report of similar aging effects at 11 and 180 cd/m^2^ luminance levels (Tobimatsu et al., 1993).

Thus, to assess the relationship between observers' retinal illuminance and their ERPs we conducted two experiments in which we recorded EEG from subjects whose retinal illuminance was manipulated using neutral density filters (Experiment 1) and pinholes (Experiment 2). Both of these methods were used previously to manipulate the amount of light that reaches observers' retinas (Eagan et al., 1999). While neutral density filters allow to control stimulus luminance, pinholes placed in front of observers' eyes act as artificial pupils altering retinal illuminance without changing stimulus luminance. Thus, the two methods complement each other and serve as a control to one another.

We tested our subjects twice to assess the reliability of our results. Our first goal was to replicate the previous finding of Rousselet et al. (2010) that aging slows down visual processing at the rate of 1 ms/year, which we successfully achieved. Further, we aimed to determine whether retinal illuminance modulates age-related delays in ERP measures of processing speed. We hypothesized that if ERP aging delays depend on senile miosis and retinal illuminance, there should be no difference in processing speed if differences in retinal illuminance are abolished. However, we found that age-related changes in processing speed are not due to senile miosis, as they were independent of luminance. Additionally, we aimed to answer whether individual differences in visual processing speed can be accounted for by variability in retinal illuminance, which they could not. Finally, by manipulating retinal illuminance in young observers, we intended, and failed, to match their ERPs to those of old observers tested at higher luminance levels. Overall, our results strongly suggest that age-related face ERP delays are not due to optical factors.

## Materials and methods

### Subjects

The study involved 59 subjects (31 females, 28 males, age range of 18–79, Table 1). To assess the test-retest reliability of the results and to control for luminance manipulation order, all but eight subjects took part in a second experimental session. Prior to the experiment, all subjects read a study information sheet and signed an informed consent form. The experiment was approved by the School of Psychology Ethics Committee (approval no. FIMS00740). We excluded persons who reported any eye condition (i.e., lazy eye, glaucoma, macular degeneration, cataract), had a history of mental illness, were taking psychotropic medications (e.g., antidepressants, beta-blockers) at the moment of testing or use to take them, suffered from any neurological condition (i.e., Parkinson's, Alzheimer's, dementia), had diabetes, had suffered a stroke or a serious head or eye injury and who had their vision tested more than 2 years ago (for people under 60 year old) or more than 1 year ago (for people aged 60 and above). Subjects' visual acuity and contrast sensitivity were assessed in the lab on the day of the first session using a Colenbrander mixed contrast card set and a Pelli–Robson chart. All subjects had normal or corrected-to-normal vision (Table 1) and contrast sensitivity in the range of 1.95 and above (normal score). One older subject reported the start of a monocular cataract that did not require medical treatment at the moment of testing. All subjects filled in a general health and life style questionnaire. All reported very good or excellent hearing and most reported at least weekly exercise. All subjects in the older group (>60) were in good cognitive health as indicated by their scores (>26 out of 30) at the MOCA test during the first experimental session. Subjects were compensated £6/h for their participation.

**Table 1:** **Subjects' information**.

**Age bracket**	**Age (median [min, max])**	**Number of subjects (females, males)**	**Visual acuity**		**MOCA scores (median, [min, max])**	**Years of education (median, [min, max])**
			**High contrast 63 cm (median [min, max])**	**Low contrast 63 cm (median [min, max])**		
18–19	19 [18, 19]	5 (4, 1)	105 [100, 110]	95 [90, 100]	n/a	15 [15, 16]
20–29	22 [21, 29]	12 (6, 6)	105 [95, 108]	94.5 [90, 102]	n/a	18 [17, 25]
30–39	32 [30, 38]	9 (2, 7)	107 [99, 109]	97 [90, 102]	n/a	18 [14, 23]
40–49	43.5 [41, 49]	8 (4, 4)	106 [95, 112]	98.5 [88, 103]	n/a	18 [12, 23]
50–59	54 [50, 59]	6 (2, 4)	105 [95, 105]	94 [90, 95]	n/a	17 [13, 19]
60–69	64.5 [60, 67]	10 (7, 3)	94 [80, 106]	85.5 [75, 95]	29 [27, 30]	15.5 [5, 21.5]
70–79	72 [70, 79]	9 (6, 3)	98 [78, 105]	88 [63, 94]	28 [26, 30]	14 [11, 21]

### Stimuli

**Figure 1:**
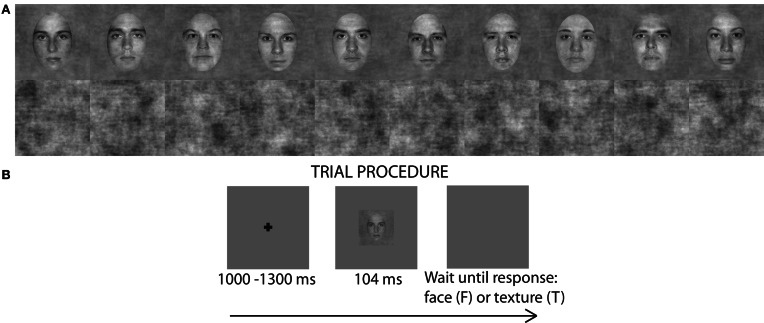
**(A)** 10 face identities at 70% phase coherence and 10 examples noise textures (0% phase coherence) used in the luminance experiment. **(B)** Single trial procedure. For presentation purposes the fixation cross and the face image are not to scale.

The stimuli were pictures of faces and textures (Figure 1A). There were 10 identities of faces (Rousselet et al., 2008). Faces were gray-scaled front view photographs oval-cropped to remove hair and pasted on a uniform gray background. A unique image was presented on each trial by introducing noise into the face images. Faces had 70% phase coherence [see details in Rousselet et al. (2008)]. Textures had random phase (0% phase coherence). All stimuli had an amplitude spectrum set to the mean amplitude of all faces. All stimuli also had the same mean pixel intensity, RMS contrast = 0.1, and occupied 9 × 9° of visual angle.

### Experimental procedure and design

Most subjects participated in two experimental sessions. The screen luminance progressively decreased in bright to dark (b2d) sessions, and increased in dark to bright (d2b) sessions. The order of the sessions was randomly assigned on the first day of testing. We altered screen luminance by placing neutral density filters in front of the computer screen. The filters were attached to thin wooden frames, which were pierced at the top, so that they could hang from pegs attached to the wall above the screen. The filters covered the screen completely. Each filter had 0.3 optical density (f-stop reduction = 2). This is equivalent to a 50% reduction in optical power transmitted through the filter. In other words, adding one filter in front of the screen reduced the screen's luminance by 50%, adding another filter reduced it by another 50% and so on.

The luminance levels from the brightest to the darkest were: 60.8, 31, 16, 8.16, 4.19, 2.17, 1.12, and 0.59 cd/m^2^. Both sessions commenced with the highest luminance block (60.8 cd/m^2^). In the b2d session, starting from block 2, the luminance was progressively reduced by adding one filter in each block to reach seven filters in block 8 (0.59 cd/m^2^). In the d2b session, in block 2 we used the maximum number of filters, seven. Then in each block we removed one filter, to reach one filter in block 8. Block 9 in each session was again conducted without any filters, as in block one (60.8 cd/m^2^). The luminance of the screen with and without filters was measured using a Minolta CS-100 colorimeter. The measurements were done at the center of the monitor about 1 h after switching it on and before running each participant.

During the experiment, subjects sat in a sound attenuated booth and rested their head on a chin rest. Stimuli were displayed on a Samsung SyncMaster 1100Mb monitor (600 × 800 pixels, height and width: 30 × 40 cm, 21 × 27° of visual angle; refresh rate—85 Hz, bits per pixel—32). Viewing distance measured from the chin rest to the monitor screen was 80 cm. Subjects were given experimental instructions including a request to minimize blinking and movement. Subjects were asked to categorise images of faces and textures by pressing “1” for face and “2” for texture, on the numerical pad of a keyboard, using the index and middle fingers of their dominant hand. Before the main experiment, subjects performed a 40 trial practice block containing 20 trials with auditory feedback, followed by another 20 trials without feedback. After the practice block, the dim lights in the booth were switched off and an adaptation screen with gray uniform background (RGB 128,128,128) was turned on. A 60 s light adaptation was performed at the beginning of all blocks, except in block 2 of d2b sessions, in which the adaptation lasted for 5 min. This longer duration was necessary due to the large luminance difference between zero and seven filters. After the adaptation and before each experimental block, pupil size in participant's right eye was measured three consecutive times (the mean of these three measurements was later used for the analyses). For the first 22 subjects we used a Colvard (Oasis Mediacla Inc.) pupillometer; for the remaining subjects we used a NeurOptics VIP™-200 pupillometer. When pupil measurement was completed, subjects were ready to proceed with the experiment.

We used a mixed design with image category and luminance as within-subject factors and age as between-subject factor. There were 9 experimental blocks, each consisting of 150 trials: 70 faces (10 face identities, each repeated 7 times, each time with a unique noise field) and 70 unique noise textures. Additionally, there were 10 practice trials (5 faces and 5 textures) at the beginning of each block with auditory feedback signaling to subjects whether their response was correct or not. The whole experiment consisted of 1350 trials, including 90 practice trials. Each trial began with a small fixation cross (size: 12 × 12 pixels; 0.4 × 0.4° of visual angle) displayed at the center of the monitor screen for a random time interval of about 1000–1300 ms, followed by an image of a face or a texture presented for about nine frames (~104 ms) (Figure 1B). These durations are multiples of refresh screen durations and do not necessarily reflect the actual duration during which image pixels were active (Elze, 2010). After the stimulus, a blank screen was displayed until subject's response. The fixation cross, the stimulus and the blank response screen were all displayed on a gray uniform background (RGB 128, 128, 128). The importance of accuracy over speed was stressed to subjects. Subjects performed the task very well: in all the luminance levels, most subjects had accuracy above 95% and all exceeded 90%. One experimental block lasted for ~4 min and the whole experiment (with breaks but excluding electrode application) lasted for about 1 h 30 min.

### EEG recording

EEG data were recorded at 512 Hz using an Active Electrode Amplifier System (BIOSEMI) with 128 electrodes mounted on an elastic cap. Four additional electrodes were placed at the outer canthi and below the eyes.

### EEG data pre-processing

EEG data were pre-processed using Matlab 2011a and the open-source toolbox EEGLAB 11.0.2.1b (Delorme and Makeig, 2004). Data were first re-referenced off-line to an average reference and an individual channel mean was removed from each channel. Data were then band-pass filtered between 0.3 and 40 Hz using a non-causal two-way least square FIR filter (pop_eegfilt function in EEGLAB). Non-causal filtering can potentially distort onsets (Vanrullen, 2011; Acunzo et al., 2012; Rousselet, 2012; Widman and Schroeger, 2012). Therefore, we analysed onsets of ERP differences by creating a second dataset in which data were pre-processed with 4th order Butterworth filters: high-pass causal filter at 2 Hz and low-pass non-causal filter at 40 Hz. Data from the two datasets were then epoched between –300 and 1200 ms around stimulus onset. Noisy electrodes were detected by visual inspection of the non-causal dataset and rejected on a subject-by-subject basis (the same electrodes were rejected in the two datasets). Baseline correction was performed using the average activity between time 0 and –300 ms. The reduction of artifacts, such as eye-movements or blinks was performed using Independent Component Analysis (ICA), as implemented in the infomax algorithm from EEGLAB. If ICA yielded components representing noisy electrodes (e.g., IC with a very focal, non-dipole activity restricted to one electrode whereas the rest of the map was flat), the noisy channels were removed and the ICA was repeated. ICA was performed on the non-causal FIR-filtered datasets and the ICA weights were then applied to the causal Butterworth-filtered datasets (on a subject by subject basis) in order to ensure removal of the same components from both datasets. After rejection of artifactual components, data were re-epoched between −300 and 500 ms and baseline correction was performed again. Finally, artifactual data epochs were removed based on an absolute threshold value larger than 100 μV and the presence of a linear trend with an absolute slope larger than 75 μV per epoch and R^2^ larger than 0.3. The median number of trials accepted for analysis was 1313 out of 1350 [min: 1163, max: 1345] in bright to dark sessions and 1318 [min: 1166, max: 1344] in dark to bright sessions.

### ERP statistical analyses

Statistical analyses were conducted in single subjects and at the group level using Matlab 2011a and the LIMO EEG toolbox (Pernet et al., 2011). To model EEG data we used a general linear model (GLM) across trials, at all-time points and all electrodes. We controlled for multiple comparisons using a bootstrap spatial-temporal clustering technique (Pernet et al., 2011; Rousselet et al., 2011; Bieniek et al., 2012).

#### Aging effects on visual processing speed

***Single subject data analyses***. We extracted several measures of visual processing speed based on the timing and the amplitude of the difference between face and texture ERPs. To that end, we used a GLM with faces and textures at each luminance level as categorical predictors. Then we computed linear contrasts (*t*-tests) between beta coefficients for faces and textures for each luminance level. This model was applied separately to the causal-filtered and non-causal filtered datasets of each subject. Thus, for each subject we obtained for every electrode the time course of model fit and of *t* statistics associated with each linear contrast. Then, for each subject, we determined the electrode with the highest squared *t* statistics in the block with the brightest luminance (60.8 cd/m^2^). It is a data-driven approach that does not make assumptions about the localization of the effects, and allows us to identify the electrode with the maximum sensitivity to our experimental manipulation, independently in each subject. We refer to this electrode as the max *t*^2^ electrode and report it according to the electrode numbering in Biosemi format (see **Figure S4** for the Biosemi electrode map with corresponding electrodes from the 10/10 system).

From the outputs of our single-subject GLMs, we derived three estimates of processing speed. The first measure was the onset of the earliest significant differences between face and texture ERPs at each luminance level. The onsets were obtained from the GLM applied to all the electrodes of the causal-filtered dataset of each subject. The second measure was the time it takes to integrate 50% of the cumulative *t*^2^ function, which we refer to as the 50% integration time (50IT) (Rousselet et al., 2010). This measure incorporates potential changes in the shape of the ERP difference waveform that may occur with age. The integration was done over time, from 0 to 500 ms, and across all electrodes. The last measure was the latency of the maximum ERP difference (peak latency) between faces and textures recorded at the max *t*^2^ electrode for each subject. Although ERP latency is not a direct index of processing speed, it could reflect the accumulation of information in neuronal population that ceases when an ERP peaks (Schyns et al., 2009). In that sense, it can potentially carry an indication of timing of neuronal processes. Both, 50IT and peak latency were obtained from the non-causal filtered data, for each luminance level and for each subject.

***Group data analyses***. To visualize age-related changes in the shape of the *t*^2^ functions (that reflects changes in the ERP difference waveform) we calculated the quantiles of the age distribution of our sample using the Harrell–Davis estimator, which is based on a weighted sum of sorted values (Wilcox, 2005). We then applied the same weights to our *t*^2^ functions for each luminance level individually (Rousselet et al., 2010).

To calculate descriptive statistics [median 50ITs with 95% confidence intervals (CI, reported in square brackets)] we used a percentile bootstrap procedure with 1000 samples and with the Harrell–Davis estimator of the median. Comparisons between 50IT for the 60.8 cd/m^2^ luminance condition and all the other luminance conditions were done using a two-tailed percentile bootstrap test for dependent groups; comparisons between young 50ITs in each pinhole condition and the 50ITs of old adults obtained in the luminance experiment (in 60.8 cd/m^2^ condition) were done using a two-tailed percentile bootstrap test for independent groups.

To determine if pupil size, retinal illuminance, our measures of processing speed (50IT, onsets, peak latencies) and peak amplitudes of ERP differences varied with age, we computed group level regressions for each luminance level using Matlab's *robustfit* function, with the default parameters. Then, we calculated percentile bootstrap confidence intervals around the slopes and intercepts in the following way. First, we sampled subjects with replacement, keeping their corresponding age, 50ITs, onsets, peak latencies and amplitudes of ERP differences. Second, we performed regressions between each measure of processing speed and age, at each luminance level. We performed these two steps 1000 times, and each time saved all the slopes and intercepts. Then, we sorted the bootstrapped slopes and intercepts, and used the 2.5 and 97.5 percentiles to form the boundaries of 95% bootstrap confidence intervals. To calculate whether the regression slopes and intercepts for the brightest condition differed from the other luminance conditions, we subtracted the bootstrapped slopes and intercepts of pairs of conditions to derive 95% bootstrap confidence intervals of the differences.

Next, we aimed to find out if, after accounting for age, we could explain individual variability in 50ITs and peak latencies of ERP differences by the variability in subjects' pupil sizes. To address that question we regressed the 50IT/age residuals and the peak latencies/age residuals against the pupil/age residuals. Again, we used a percentile bootstrap procedure to build confidence intervals of the slopes and intercepts.

Finally, we determined the onset and maximum latency of the aging effects by calculating how much of the cumulative *t*^2^ function of each subject has been integrated up to each time point between 0 and 500 ms. Then, at each time point, and for each luminance level separately, we calculated regressions across subjects between the integrated *t*^2^ and age. We determined when the regression slopes became significantly different from zero using a bootstrap procedure (see Rousselet et al., 2010 for description).

#### Luminance effect on face-texture ERP differences

***Single subject data analyses***. In the second part of the analyses, we quantified the time course of luminance effects on face and texture ERPs using a single-trial ANCOVA model. The model had two categorical predictors—faces and textures, one continuous predictor—luminance, and an interaction term between luminance and category. Luminance was entered into the model as the z-score of the log luminance levels. This model was applied to causal and non-causal filtered datasets of individual subjects. From the analyses of the causal-filtered datasets we obtained onsets of luminance and luminance × category interactions. From the analyses of the non-causal filtered data we obtained the latency of the strongest luminance and interaction *F* values.

***Group data analyses***. To determine if the ERP onsets and the latencies of maximum sensitivity to luminance and luminance × category change with age, we regressed the onsets and maximum latencies of each effect against age. Then, we calculated 95% bootstrap confidence intervals around the slopes and intercepts as well as around the difference between the slopes and intercepts of the two effects. We used a similar procedure as described in section Group data analyses.

#### Overlap between the ERPs of young and old observers

In the third part of our analyses, we determined if we could match the ERPs of old observers in the brightest condition, to that of young observers at lower luminance levels. To this end, we quantified the overlap between the *t*^2^ functions of older (>60, *n* = 18) observers in the brightest luminance condition and young observers (<30, *n* = 15) at each luminance level. First, we normalized the *t*^2^ functions within participant by dividing their *t*^2^ functions by the maximum *t*^2^ across all luminance levels and time points. Then, we averaged the *t*^2^ functions across subjects, separately for young and old subjects. To calculate the percentage of *t*^2^ overlap between young and old, we computed the area under the mean *t*^2^ functions for young and old observers using trapezoidal numerical integration (*trapz* function in Matlab) and expressed it as a proportion of the overall area under the two functions. The overlaps where calculated between the mean *t*^2^ function of young subjects at each luminance level and the mean *t*^2^ function of old subjects in the two conditions with the highest luminance (60.8 cd/m^2^—conditions 1 (c1) and 9 (c9)).

The 95% confidence intervals around the overlaps as well as around mean *t*^2^ functions of young and old adults were computed using a bootstrap procedure. First, separately for the young and old group, we sampled subjects with replacement. We then computed mean *t*^2^ functions for young and old samples and calculated the overlap between the two functions for each luminance level. We performed this procedure 1000 times, each time saving the mean *t*^2^ functions for young and old groups in each condition, the overlaps and the difference in overlaps between the two brightest conditions (the first and the last block). We then sorted each of the bootstrap estimates and used the 2.5 and 97.5 percentiles to form the boundaries of 95% bootstrap confidence intervals. We also computed within-old-group overlaps by sampling with replacement two samples of old subjects, and calculating the overlap between their means following the same procedure we used for between-group overlaps. Within-young-group overlaps were obtained using the same approach.

## Results

The first goal of this study was to replicate the ERP aging effects reported in Rousselet et al. (2010). Second, we aimed to determine if age-related delays in ERP measures of visual processing speed are luminance dependent. Third, we set to answer whether individual differences in processing speed can be explained by the variability in observers' retinal illuminance. Finally, we wanted to determine if the ERPs of old observers can be matched to the ERPs of young observers tested at lower retinal illuminance levels.

**Figure 2:**
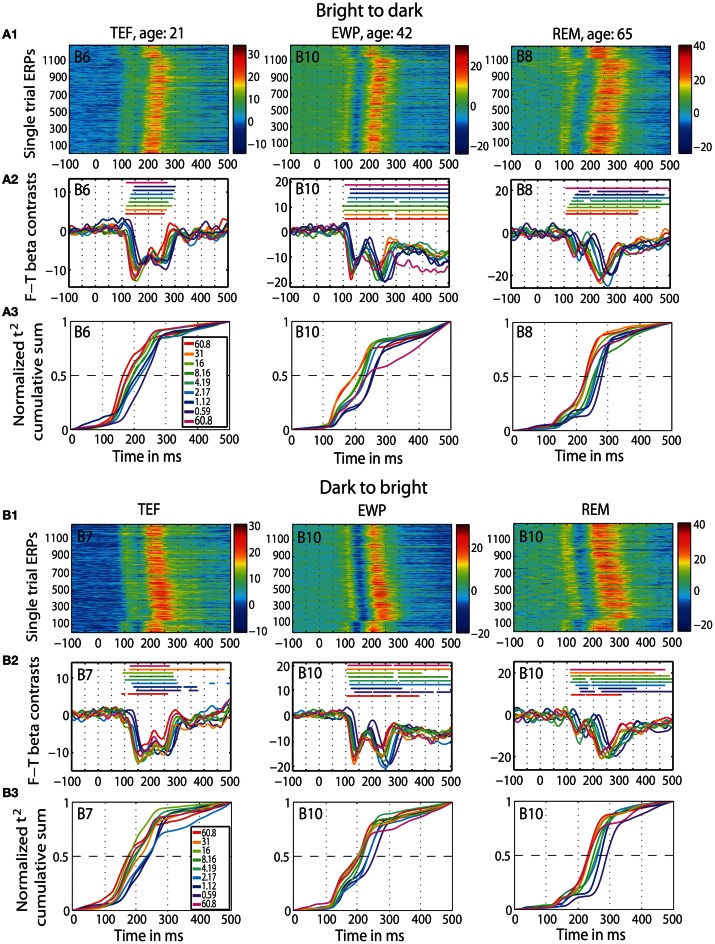
**ERP results in individual subjects.** Data of three representative subjects: one young (TEF, age 21), one middle-age (EWP, age 42) and one old (REM, age 65) for b2d session (panel **A**) and d2b session (panel **B**) at the max *t*^2^ electrode (indicated in the top left corner of each subplot). An electrode map is provided in **Figure S4**. (**A1** and **B1**) Single trial ERPs, in μV. (**A2** and **B2**) Time-courses of contrasts between face and texture beta-coefficients for each luminance level; horizontal lines indicate significant contrasts. (**A3** and **B3**) Normalized *t*^2^ cumulative sums at each luminance level.

First, we replicated previous findings of Rousselet et al. (2010): aging slows down visual processing, expressed in the 50IT, at the rate of 1 ms/year. We observed this delay at all luminance levels, which suggests that age effects are not luminance dependent. We also found that aging prolongs peak latencies of the face-texture ERP differences at the average rate of 1.5 ms/year. However, we found no effects of age on the onset of ERP differences. We also found that early ERPs to faces and textures were delayed with decreasing luminance, an effect visible in individual subjects in both sessions (Figure 2). Finally, we were unable to explain individual differences in visual processing speed by inter-subject variability in retinal illuminance. We also did not manage to match the ERPs of old observers to those of young adults at lower luminances. These findings suggest that the age-related visual slowdown is not due to optical factors.

### Age effects on 50% integration times, peak latencies, onsets and amplitudes of face-texture ERP differences

**Figure 3:**
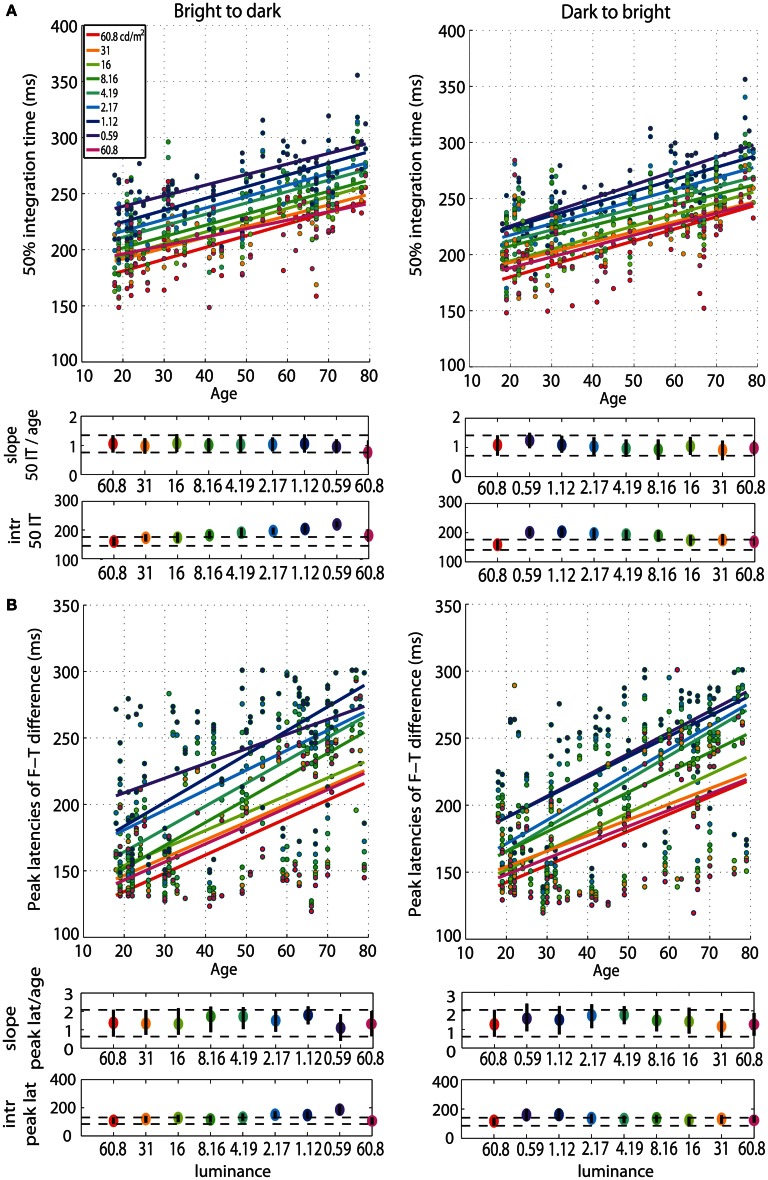
**Regressions of 50IT and peak latencies of face-texture ERP differences against age. (A)** Regression fits between 50IT and age and **(B)** latencies of maximum face-texture ERP differences, for all luminance levels. B2d sessions are shown in column 1, d2b sessions in column 2. The two horizontal plots below each regression plot contain slopes and intercepts (intr) as colored dots, with confidence intervals as vertical black lines. Horizontal dashed black lines show the boundaries of the confidence intervals of the slopes and intercepts in the first brightest condition (60.8 cd/m^2^).

First, we observed a qualitative age-related change in the overall shape of the *t*^2^ functions for all luminance levels (**Figure S1**). This qualitative change was captured by our measure of processing speed (50IT), showing a significant age-related delay of ~1 ms/year (Figure 3A, **Tables S1, S2**). This effect was present at all luminance levels, and in both experimental sessions. There was no significant difference between the 50IT/age regression slope at the brightest luminance level (60.8 cd/m^2^) and at all the other luminance conditions, suggesting that the 1 ms/year slope is similar across luminance levels (**Tables S1–S4**). The peak latencies of face-texture ERP differences were also delayed by age at all luminance levels and in both sessions, with an average slope of 1.5 ms/year (Figure 3B, **Tables S1, S2**).

**Figure 4:**
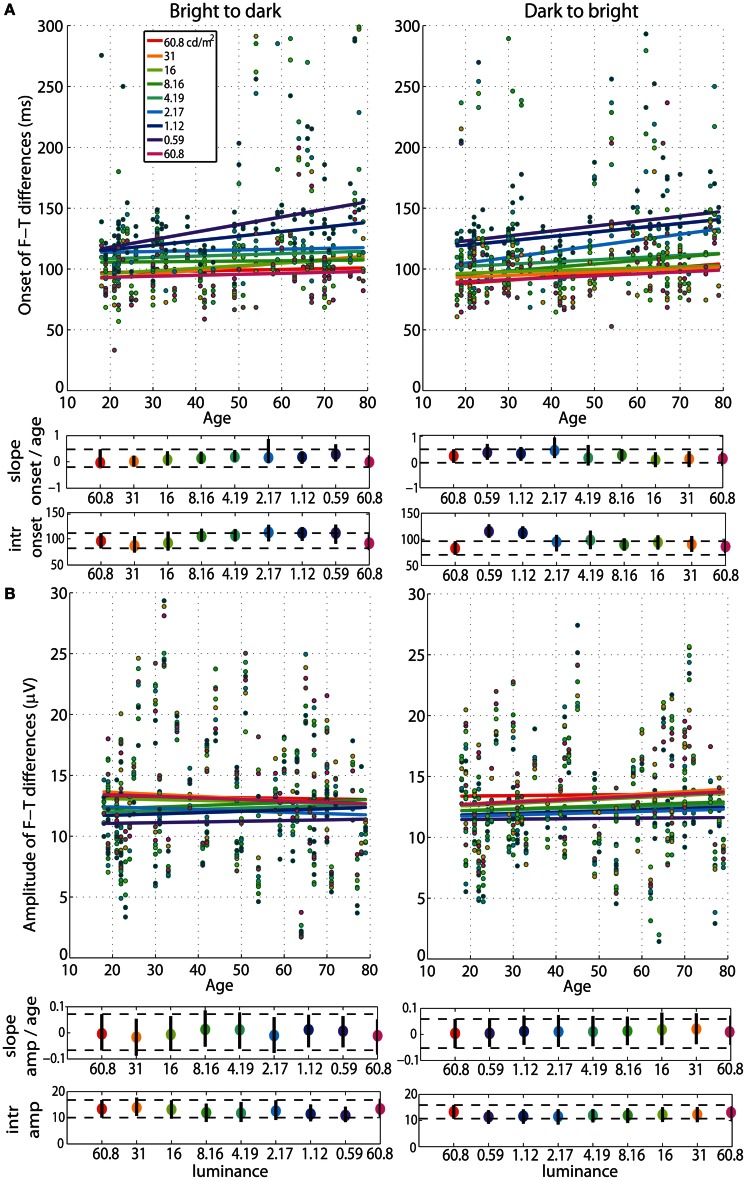
**Regressions of onset and amplitude ERP differences against age. (A)** Regression fits between the onset of significant face-texture ERP differences and age and **(B)** between maximum amplitude of face-texture ERP differences and age, for all luminance levels.

Our analyses revealed no age effect on the onsets of face-texture ERP differences, except for some small effects that were inconsistent across sessions, present at 0.59 cd/m^2^ in the bright to dark session only, and at the 60.8, 1.12, and 2.17 cd/m^2^ in the dark to bright session only (Figure 4, **Tables S1, S2**). We also found no aging effect on the amplitude of face-texture ERP differences at all luminance levels and in the two experimental sessions (Figure 6, **Tables S1, S2**).

**Figure 5:**
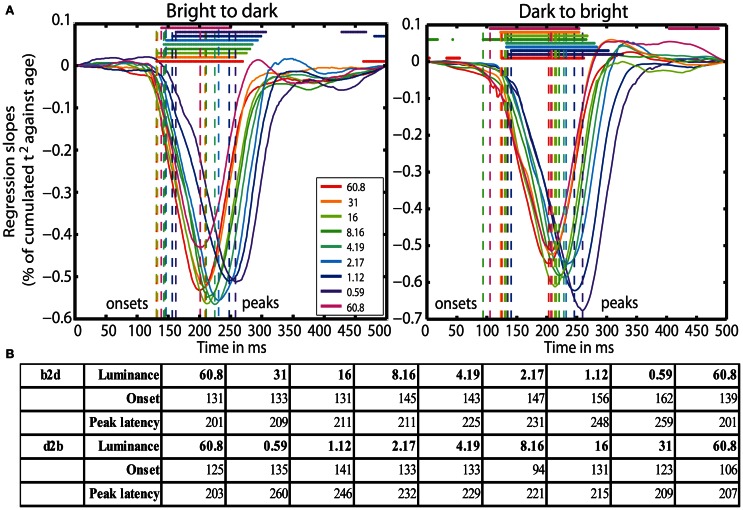
**(A)** Time-courses of % of cumulated *t*^2^/age regression slopes. Each curve shows the time-course at one luminance level. Horizontal lines indicate significant regression slopes. Vertical dashed lines mark the onsets and the peak latencies of significant aging effects. **(B)** Table of onsets (ms) and peak latencies (ms) of the aging effects on processing speed for all luminance levels (cd/m^2^) for b2d and d2b experimental sessions.

Finally, aging started to affect processing speed at 131 ms (b2d) and at 125 ms (d2b) post-stimulus at 60.8 cd/m^2^, except in blocks 7 (8.16 cd/m^2^) and 9 (60.8 cd/m^2^) of the d2b session, where aging effects commenced already at 94 and 106 ms, respectively. Aging effects were the strongest around 201 ms (b2d) and 203 ms (d2b) at the highest luminance and were delayed up to ~260 ms at 0.59 cd/m^2^. Reduced luminance also prolonged the onset of aging effects from ~125 ms at 60.8 cd/m^2^ up to 162 ms at 0.59 cd/m^2^ (Figures 5A and 5B).

### Age effects on pupil size and retinal illuminance

**Figure 6:**
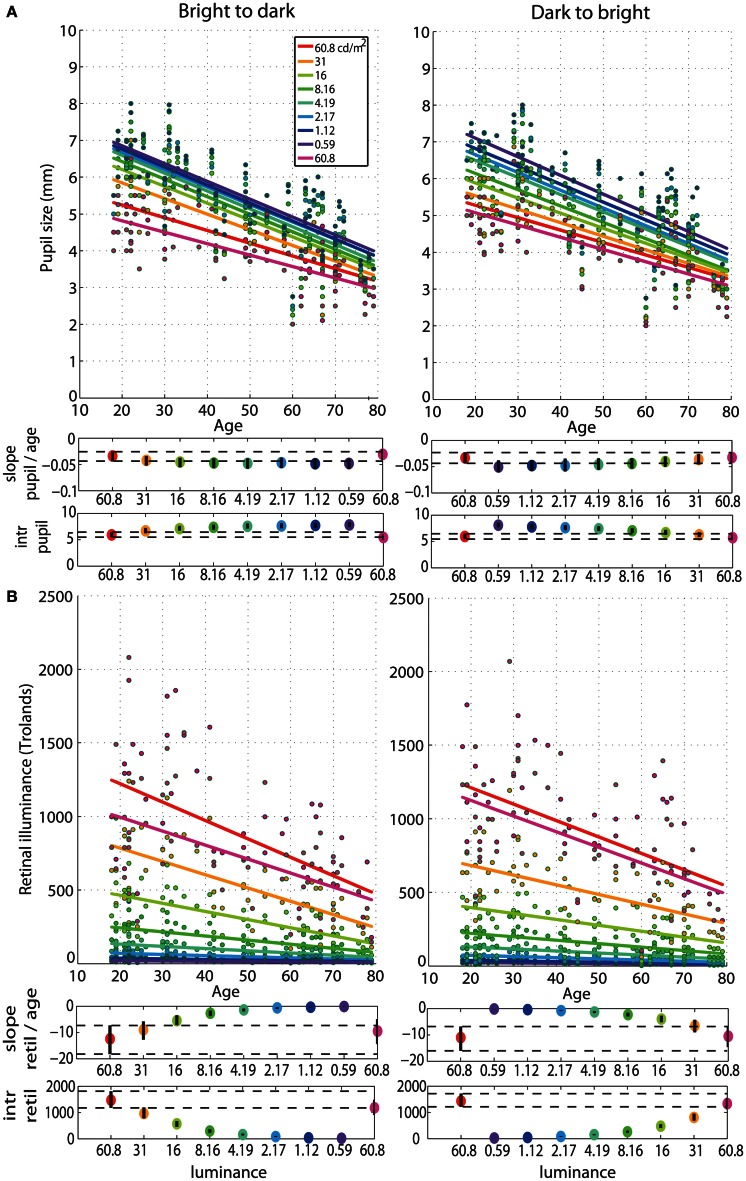
**Regressions of pupil size and retinal illuminance against age. (A)** Regression fits between pupil size and age and **(B)** between retinal illuminance (retil) and age, for all luminance levels. See Figure 4 caption for details.

The regressions between pupil size and age revealed senile miosis—a significant reduction of pupil size with age that was present at all luminance levels in both sessions (Figure 6). The slope of the pupil/age regression was in the range of −0.03 mm (60.8 cd/m^2^) to −0.05 mm (0.59 cd/m^2^) per year (**Tables S1, S2**). This is equivalent to about 0.6–1 mm decrease in pupil size every 20 years—from ~5 mm at 20 years old to ~3.5 mm at 80 years old at 60.8 cd/m^2^ and from ~7 mm at 20 years old to ~4.5 mm at 80 years old at 0.59 cd/m^2^. The pupil/age regression slope at the brightest luminance did not differ from the slopes at the lower luminance levels (**Tables S1, S2**). The intercepts of the pupil/age regressions differed between the brightest luminance and all the other conditions and increased from 5 mm at 60.8 cd/m^2^ to 7 mm at 0.59 cd/m^2^.

As expected, retinal illuminance decreased with increasing age (Figure 6) and both the slope and the intercept of the retinal illuminance/age regression differed significantly between 60.8 cd/m^2^ and all the other luminance conditions (**Tables S1, S2**). The slope ranged from about −12 at 60.8 cd/m^2^ to −0.2 at 0.59 cd/m^2^ in both sessions. The intercept was 1400 Td at 60.8 cd/m^2^ and dropped to 24 Td at 0.59 cd/m^2^.

**Figure 7:**
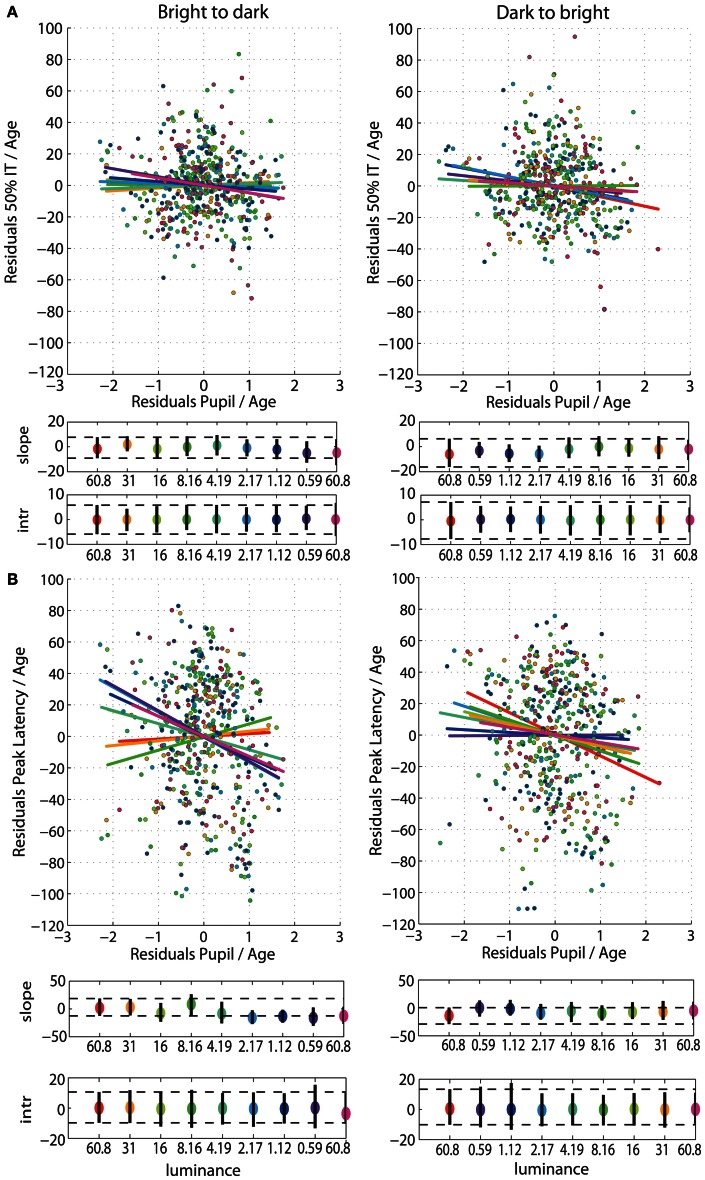
**(A)** Regressions of residuals of 50IT/age against residuals of pupil size/age for all luminance levels. **(B)** Regressions of residuals of peak latency of face-texture ERP differences/age against residuals of pupil size/age for all luminance levels. See Figure 4 caption for details.

After partialling out the effect of age from 50IT, from peak latencies of face-texture ERP differences and from pupil size, individual differences in 50IT (Figure 7A) and in peak latencies (Figure 7B) could not be accounted for by variability in pupil size across subjects (**Table S5**). Regression slopes between 50IT/age residuals and pupil size/age residuals were not significant at any luminance level.

### Age effects on ERP sensitivity to luminance and category × luminance interaction

**Figure 8:**
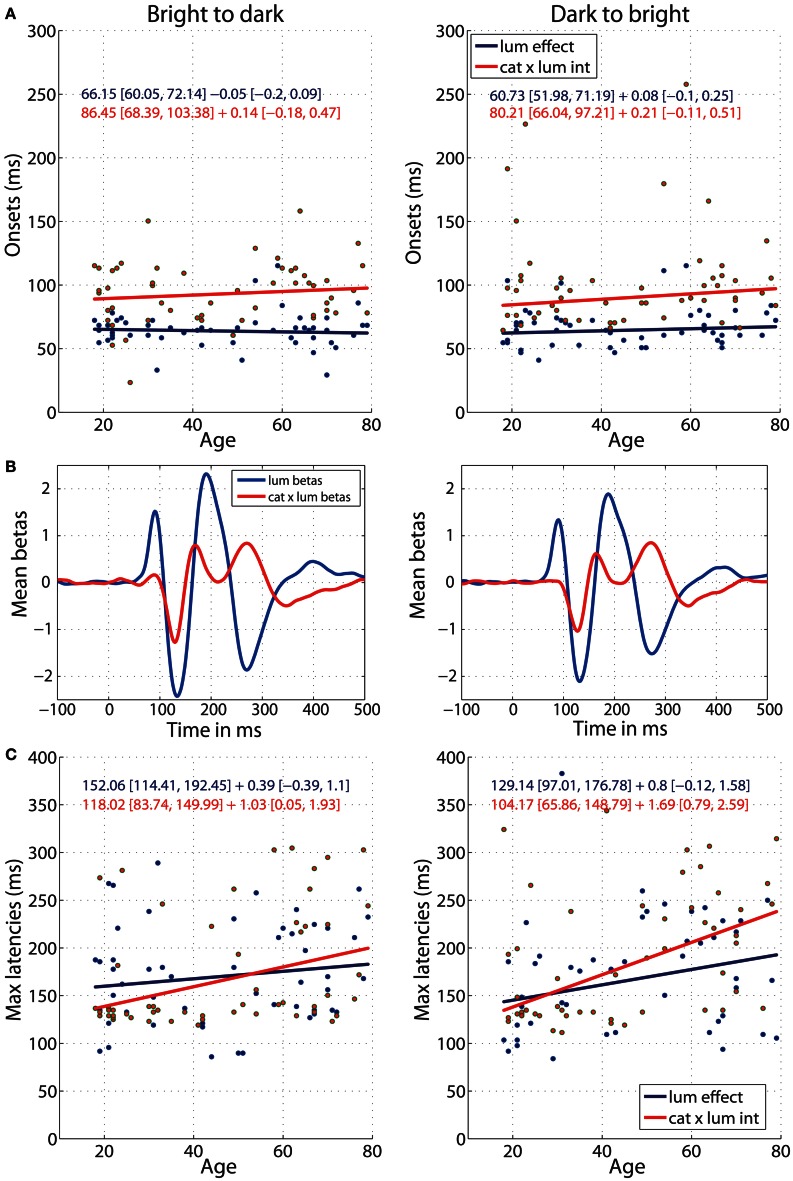
**Regressions of luminance effect and category × luminance interaction against age. (A)** Onsets of the two effects against age for b2d (left column) and d2b sessions (right column). **(B)** Mean (across all subjects) beta coefficients associated with two predictors: luminance and category × luminance interaction. **(C)** Latencies of the maximum effects against age. Each subplot contains regression equations in the format intercept + slope with their confidence intervals in square brackets. The color of each equation corresponds to the regression line for each effect.

The onsets of ERP sensitivity to luminance and to category × luminance interaction did not change with age (Figure 8A) and the age regression slopes for the two effects did not differ (b2d: diff = −0.19 [−0.51, 0.10]; d2b: diff = −0.13 [−0.42, 0.15]). Luminance started to affect the ERPs at about 66 ms [60, 72] in the b2d session and 60 ms [52, 71] in d2b session (Figure 8A). This is about 20 ms before (b2d: diff = −20 ms [−36 −3]; d2b: diff = −19 ms [−34, −4]) luminance began to interact with stimulus category at 86 ms [68, 103] (b2d) or 80 ms [66, 97] (d2b) (Figures 8A,B). The ERP sensitivity to luminance was the strongest around 152 ms in b2d session and 129 ms in d2b session, whereas the category × luminance interactions peaked at about 118 ms (b2d) and 104 ms (d2b) post-stimulus (Figures 8B,C). However, the latencies of the two effects did not differ significantly (differences between regression intercepts, b2d: diff = 34 [−9, 84]; d2b: diff = 25 [−32, 91]). There was also no age effect on the timing of maximum sensitivity to luminance in any of the sessions (Figure 8C). However, aging delayed the latency of maximum interaction between stimulus category and luminance at the rate of 1.03 ms [0.05, 1.93] per year in b2d and 1.69 ms [0.79, 2.59] per year in d2b. The difference between the regression slopes of luminance and category × luminance effects was not significant (b2d: diff = −0.64 [−1.83, 0.69]; d2b: diff = −0.89 [−2.17, 0.26]).

### Overlap between young and old subjects

**Figure 9:**
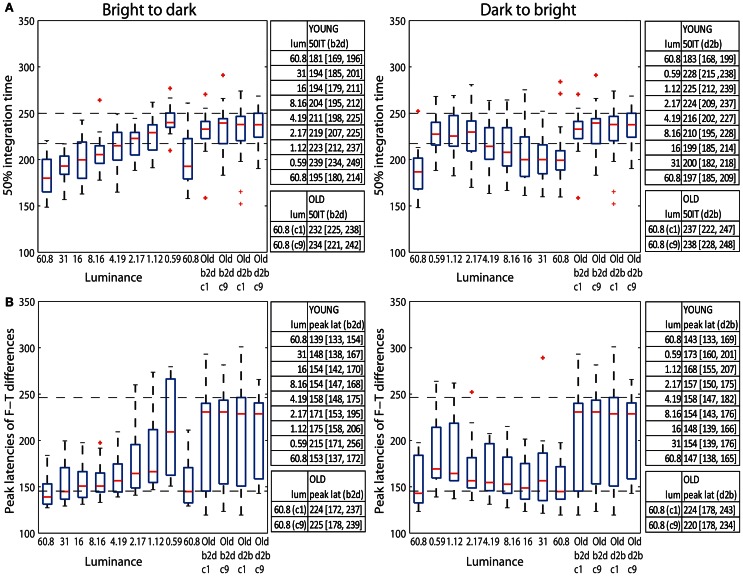
**(A)** Boxplots of 50% integration times (ms) and **(B)** peak latencies (ms) of face-texture ERP differences for young (<30 years old) and old (>60 years old) adults. The median (across subjects) 50ITs and peak latencies along with confidence intervals are provided in the tables to the right of the subplots. For young subjects median 50IT and peak latencies for all conditions are given; for old subject, they are given for the two brightest conditions [condition 1 (c1) and 9 (c9)].

The median 50IT of young adults (<30) at the highest luminance (60.8 cd/m^2^) was 181 ms (95% bootstrap CI = [169, 196] in b2d) and 183 ms [168, 199] in d2b (Figure 9A tables). At the same luminance, the median 50IT of older subjects (>60) was 232 ms [225, 238] (b2d) and 237 ms [222, 247] (d2b), which is ~50 ms slower than the processing speed of young subjects in both experimental sessions (b2d: diff = −50 ms [−64, −34]; d2b: diff = −53 ms [−72, −33]). Indeed, visual processing of young adults was significantly faster than that of old adults in all but the two darkest conditions (1.12 and 0.59 cd/m^2^) in both sessions, and the 2.17 cd/m^2^ condition in d2b session (**Table S6**).

The latencies of maximum face-texture ERP differences were also delayed by age in both sessions. ERP differences in young adults at the luminance of 60.8 cd/m^2^ peaked at the median latency of 139 ms [133, 154] (b2d) and at 143 ms [133, 169] (d2b), whereas for old adults the differences peaked at 224 ms [162, 238] (b2d) and at 224 ms [175, 242] (d2b) (Figure 9B tables). This is an ~80 ms difference between peak ERP latencies of young and old subjects at the highest luminance (b2d: diff = −84 ms [−100, −33]; d2b: diff = −80 ms [−100, −27]). The latencies of the peak ERP differences of old adults (at 60.8 cd/m^2^) were significantly longer than those of young adults for all luminance levels, apart from 1.12 and 0.59 cd/m^2^ conditions in both sessions and 2.17 cd/m^2^ in b2d session only (**Table S6**).

**Figure 10:**
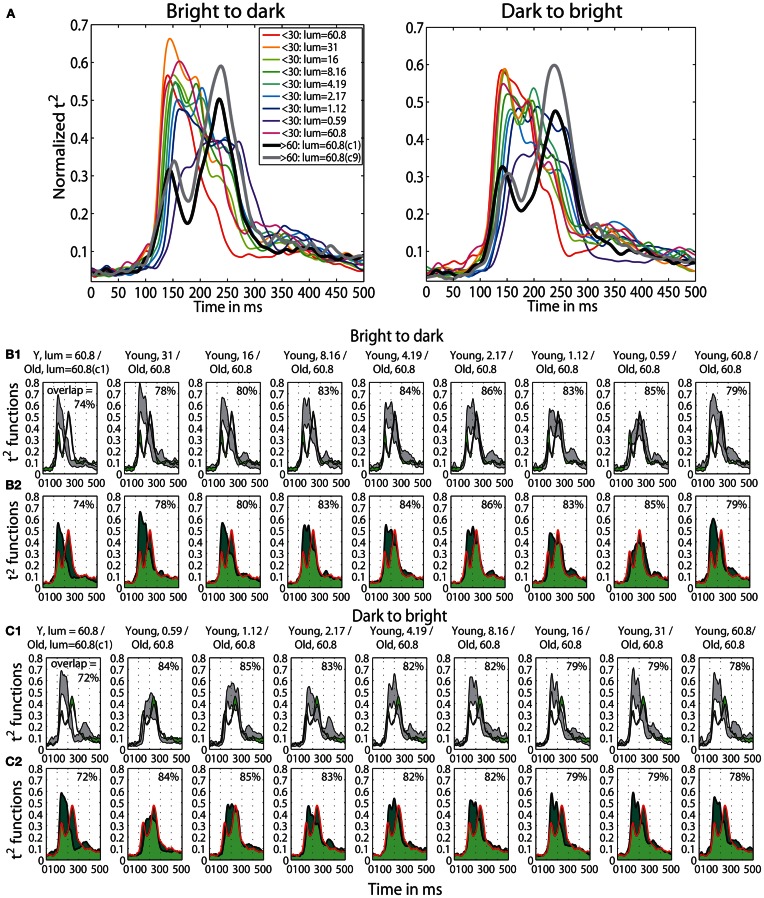
**(A)** Time-course of normalized *t*^2^ functions for young and old subjects. Each subplot shows mean time-courses, across young subjects (<30 years old), at each luminance level, and across old subjects (>60 years old) in the two conditions with the highest luminance: condition 1 (c1) plotted in black and condition 9 (c9) plotted in gray. **(B1,C1)** Confidence intervals of young and old *t*^2^ functions. Each subplot shows the time-course of 95% confidence intervals of the mean *t*^2^ functions of young subjects (in gray) at each luminance level and of old subjects (in green) in the first brightest condition (luminance = 60.8 cd/m^2^). **(B2,C2)** Overlaps between young and old *t*^2^ functions. Each subplot depicts the area under the *t*^2^ function of young (<30) and old (>60) subjects shaded in dark green. The edges of the young *t*^2^ functions are black, those of old *t*^2^ functions are red. The overlap between *t*^2^ functions for young and old subjects is shaded in light green. The proportion of overlap is given inside each subplot and the luminances are given in the title of each subplot.

**Figure 11:**
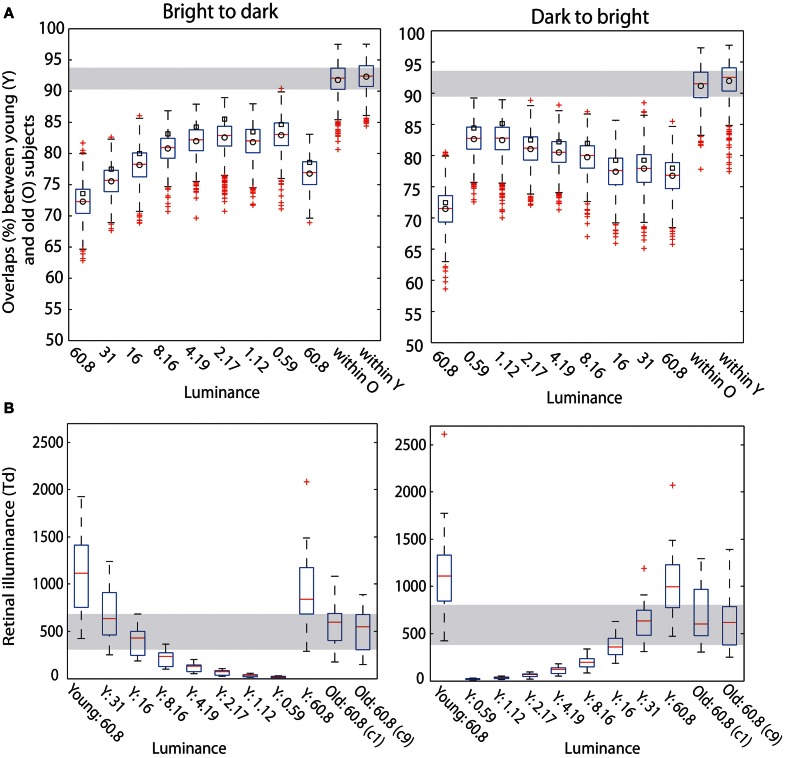
**(A)** Boxplots of overlaps. Boxplots of bootstrapped *t*^2^ function overlaps between young and old adults. In each subplot, the first nine boxplots show overlaps (%) between young subjects in each luminance level and old subjects in the first brightest luminance condition (c1, lum = 60.8 cd/m^2^). The last two boxplots show the within group overlaps for old and young subjects, in the first brightest condition (60.8 cd/m^2^) for b2d and d2b session. In each boxplot the square indicates the percentage of overlap between young and old yielded by our calculation; the circle is the mean of the bootstrapped overlaps—thus, the difference between the values for circle and square suggests that our estimation of the overlap is positively biased. **(B)** Retinal illuminance of young and old subjects. The first nine boxplots in each subplot depict the distributions of retinal illuminances in young subjects, at nine luminances; the two last boxplots in each subplot show results in old subjects in the two brightest conditions (luminance = 60.8 cd/m^2^).

A qualitative age-related change in the shape of t^2^ functions was shown in Figure 2; Figure 10A shows the mean *t*^2^ functions for young (<30 years old) and old (>60 years old) adults. We computed the percentage of overlap between normalized *t*^2^ functions of these two age groups to determine if the brain responses of old adults could be matched to that of young adults experiencing reduced retinal illuminance. The overlap increased with decreasing luminance, starting from about 69–74% at 60.8 cd/m^2^ to about 83–86% at 0.59 cd/m^2^ in both sessions (Figures 10B,C, 11A). The overlap within the old group exceeded 90% indicating that old subjects are more similar to each other than to young subjects at any luminance level (Figure 11A). In addition, even in the two conditions where the overlap was the highest—86% at 0.59 cd/m^2^ and 85% at 1.12 cd/m^2^, the retinal illuminance of young subjects was only about 5% of that of old subjects (Figure 11B), thus suggesting that retinal illuminance cannot account for young vs. old differences in processing speed.

## Pinhole experiment

To investigate if there is a causal relationship between pupil size and processing speed, we conducted a second experiment in which young subjects were wearing pinholes ranging from 1 to 5 mm. We aimed to establish if we could match old subjects' processing speed at high luminance (60.8 cd/m^2^) to that of young subjects wearing pinholes.

## Methods

### Subjects

10 subjects (median age = 28.5, min. = 22, max. = 34, six males, 10 right handed) took part in two experimental sessions conducted 1 week apart. Seven of them also participated in the luminance experiment 5–6 months earlier. Each subject's visual acuity and contrast sensitivity were measured for both eyes separately (monocular testing), on the day of the first session, using the same materials as in the luminance experiment. All subjects had normal or corrected-to-normal vision and contrast sensitivity (Table 2), and all reported very good hearing, at least weekly exercise and none reported smoking. None of the subjects reported suffering from an eye disease, or a mental condition and none was taking psychotropic medications. All subjects gave written informed consent and were compensated for their participation at the rate of £6/h.

**Table 2:** **Pinhole experiment subjects' information**.

**Visual acuity**						
**High contrast 63 cm (median [min, max])**		**Low contrast 63 cm (median [min, max])**		**Contrast sensitivity (median [min max])**		**Years of education (median [min max])**
**Right eye**	**Left eye**	**Right eye**	**Left eye**	**Right eye**	**Left eye**	
107 [89, 109]	105.5 [98, 112]	95 [88, 99]	93 [89, 99]	1.95 [1.95, 2.10]	1.95 [1.95, 2.10]	19 [15, 25]

### Stimuli

The stimuli were faces and textures generated as in the luminance experiment.

### Experimental design

The experiment consisted of seven blocks in each session. Pinhole order differed in the two experimental sessions: in the “small to big” (s2b) session, a 1 mm pinhole was applied in block 2. Pinhole size then increased by 1 mm in each subsequent block to reach 5 mm in block 6. In the “big to small” (b2s) session, a 5 mm pinhole was used in the 2nd block and then pinhole size decreased by 1 mm in each block, up to 1 mm in block 6. The first and the last blocks in both sessions were conducted without any pinholes. All subjects participated in one s2b and one b2s session that were randomly assigned.

Each block contained 210 trials: 100 faces (10 face identities repeated 10 times, each time with unique noise field), 100 unique noise textures, and 10 practice trials at the beginning of every block (five faces and five textures). The whole experiment had a total of 1470 trials. The task and trial procedure were the same as in the luminance experiment.

### Procedure

The experiment was conducted in the same lab booth as the luminance experiment. The stimuli were displayed on the same monitor with a luminance of 60.8 cd/m^2^, which was constant across blocks. The viewing distance was also 80 cm. Subjects performed the experiment monocularly using the eye with best visual acuity—four subjects used their left eye and six subjects used their right eye. The other eye was occluded with an optician eye patch. For the purpose of light adaptation, before each experimental block, subjects were instructed to look at the monitor screen with uniform gray background (128 128 128) and luminance of 60.8 cd/m^2^ for 60 s. After adaptation, subjects' pupil size in the non-occluded eye was measured using a NeurOptics pupillometer, following the same procedure as in the luminance experiment. After pupil measurement, an optical trial lens frame (model TF-1002, Danyang Huasu Optical Co., Ltd.) was put on subjects' head. The pinholes were black circular plates 4 cm in diameter with a circular aperture of 1, 2, 3, 4, or 5 mm located in the middle of the plate. To determine the optimal position of the pinhole in front of the non-occluded eye, the smallest (1 mm) pinhole plate was inserted into the trial frame. Subsequently, the experimenter adjusted the pinhole position until a rectangular frame displaying the message “Press any key to start … (Block 1 of 7)” (size: 256 × 256 pixels, 9° × 9° of visual angle, displayed in the center of the screen) was centered in the subjects' visual field (the message was not displayed during light adaptation). Each subject's visual field extent, while looking through the pinhole, was computed by taking into consideration the distance between the eye and the pinhole plate and the pinhole size. The median visual angle across subjects for pinholes of 1, 2, 3, 4, or 5 mm was 15, 17, 18, 20, and 22°, respectively, in the s2b and 14, 16, 18, 20, and 22° in the b2s procedure. Thus, the 9 × 9° stimuli were visible even through the smallest pinhole. Once the trial frame with 1 mm pinhole was optimally installed, subjects conducted a 40 trial practice block, which was similar to the practice block in the luminance experiment. After the practice block and a small break, subjects proceeded with the experiment.

### EEG data acquisition and pre-processing

Data were acquired and pre-processed as in the luminance experiment, except that we did not create causal-filtered datasets. All analyses were done on the non-causal filtered data (band-pass filtered between 0.3–40 Hz using a two-way least square FIR filter (pop_eegfilt function in EEGLAB).

### EEG data analysis

EEG data were analysed using Matlab 2011a and the LIMO EEG toolbox (Pernet et al., 2011). We used general linear modeling of single-trial EEG data and the procedure was similar to the one used in the luminance experiment, except there were seven face-texture contrasts, instead of nine—one for each of the seven pinhole conditions. As in the luminance experiment, the results were corrected for multiple comparisons using a spatial-temporal clustering approach.

For descriptive statistics and all comparisons, we used percentile bootstrap procedures as in the luminance experiment.

## Results

**Figure 12:**
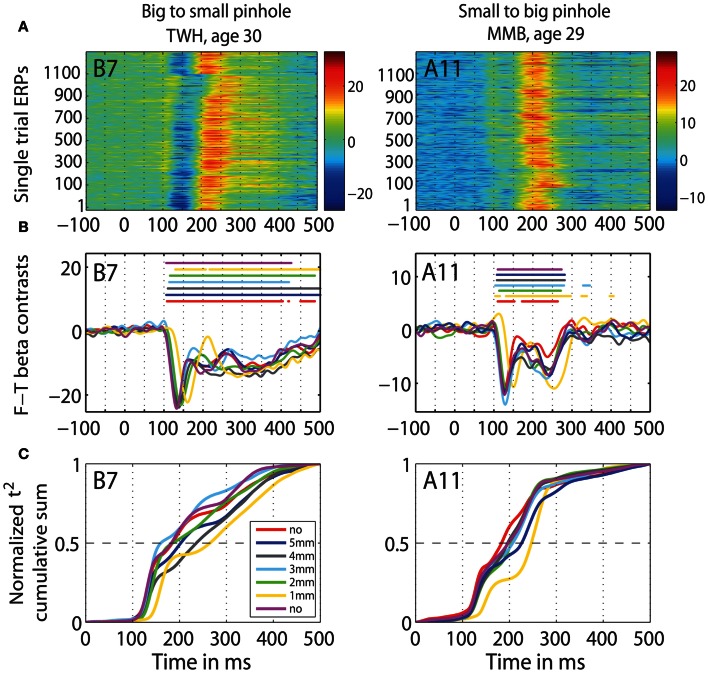
**Individual subjects data.** Data of two representative subjects: TWH (age 30), and MMB (age 29) in b2s (column 1) and s2b (column 2) sessions at the max *t*^2^ electrode for that subject, indicated in the top left corner of each plot. **(A)** Single-trial ERPs. **(B)** Time-courses of contrasts between face and texture beta-coefficients for each pinhole condition. Horizontal lines indicate significant differences. **(C)** Cumulative sums of *t*^2^ functions for each pinhole condition.

The goal of the second experiment was to determine if, by decreasing young subjects' pupil size, we could slow their processing speed and match their ERPs to those of old subjects. We found that the ERPs of young subjects were delayed for all pinhole sizes compared to the no pinhole condition. This effect was the strongest with the 1 mm pinhole and was visible at the level of single trial ERPs (Figure 12A), face-texture ERP differences (Figure 12B), and cumulative sums of *t*^2^ functions (Figure 12C). Also, contrary to our hypothesis that the smaller the pinhole, the bigger the overlap between young and old subjects' ERPs, the overlap was higher for 4 and 5 mm pinholes compared to 1 and 2 mm pinholes (Figure 15). However, even at 4 or 5 mm, we were unable to match the ERPs of young observers to those of old observers.

### Effect of pinholes on ERP processing speed

**Figure 13:**
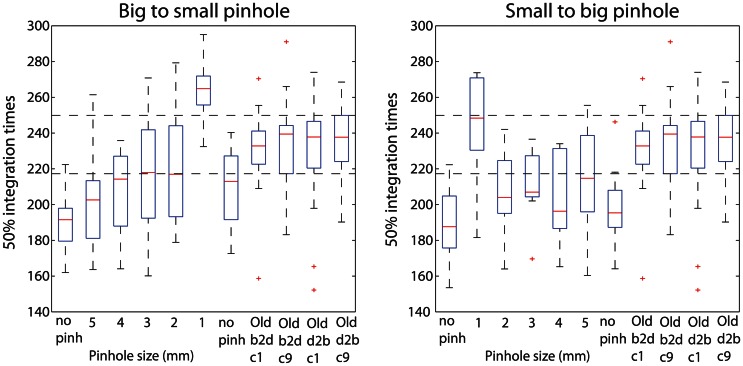
**Boxplots of 50% integration times.** Boxplots 1–7 show 50ITs across all pinhole subjects, in each pinhole condition. Boxplots 8–11 show old subjects' 50ITs from the two brightest conditions of the luminance experiment (60.8 cd/m^2^—c1 and c9), for the b2d and d2b sessions. Horizontal dashed lines indicate the lowest 25th and the highest 75th quantile across the old subjects' four conditions.

Young subjects' 50ITs increased from 189 ms in the first no pinhole condition to 265 ms (b2s) and 251 ms (s2b) with a 1 mm pinhole (Figure 13, Table 3). We found significant differences in 50ITs between the no pinhole condition and 1, 2, and 3 mm pinhole sizes in both sessions; for 4 mm the difference was significant only in the b2s session, and for 5 mm pinhole in the s2b session. There was no significant difference between the two no pinhole conditions within the same session.

**Table 3:** **50% integration times (50IT) for all pinhole conditions**.

	**No pinhole (first)**	**1 mm pinhole**	**2 mm pinhole**	**3 mm pinhole**	**4 mm Pinhole**	**5 mm pinhole**	**No pinhole (last)**
b2s session	189 [177, 204]	265 [251, 277]	219 [194, 248]	219 [191, 243]	211 [187, 227]	198 [180, 216]	211 [190, 226]
	Difference	−75 [−88, −62]	−29 [−57, −10]	−29 [−54, −4]	−22 [−38, −3]	−9 [−24, 5]	−22 [−40, 2]
s2b session	189 [174, 205]	251 [216, 267]	205 [193, 228]	210 [200, 225]	204 [186, 226]	217 [195, 237]	196 [180, 212]
	Difference	−62 [−73, −42]	−16 [−28, −5]	−20 [−30, −13]	15 [−35, 1]	−27 [−47, −5]	−7 [−27, 15]

The median 50IT (ms) and its 95% CI (in square brackets) is given for each pinhole condition and each session. Differences between the first “no pinhole” condition and each of the remaining pinhole conditions (1−5 mm and the last condition with “no pinhole”) are also provided along with the 95% CIs.

### Matching of processing speed between young and old subjects

Young subjects' 50ITs for 2 and 3 mm pinholes in the b2s session matched those of old subjects from both b2d and d2b luminance sessions (b2d: 2 mm—diff = −14 [−39, 16]; 3 mm—diff = −14 [−41, 11]). However, in the s2b session, 50IT matched between young and old subjects (from both luminance sessions) for 1 and 5 mm pinhole sizes (b2d: 1 mm—diff = 19 [−15, 36]; 5 mm—diff = −16 [−38, 5]; d2b: 1 mm—diff = 14 [−16, 37]; 5 mm—diff = −20 [−43, 1]). 50ITs for all the remaining pinhole sizes differed significantly from those of old subjects (Figure 13, **Table S7**).

**Figure 14:**
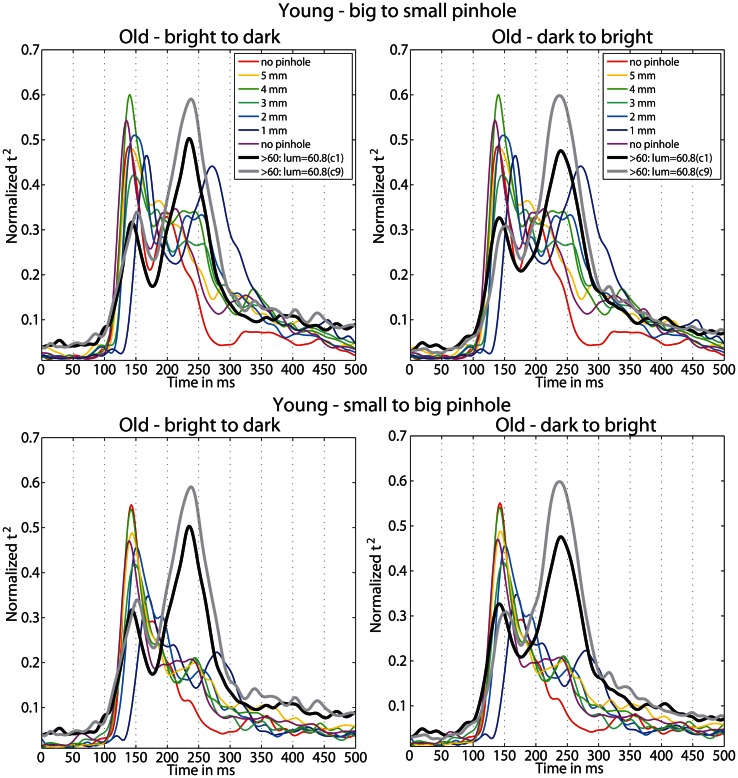
**Time-course of normalized *t*^2^ functions for young and old subjects.** Each subplot shows the time-courses of mean normalized *t*^2^ functions of young subjects, in all conditions of the pinhole experiment, and of old subjects from the luminance experiment, in the two conditions with the highest luminance 60.8 cd/m^2^: condition 1 (c1) plotted in black, and condition 9 (c9) plotted in gray.

**Figure 15:**
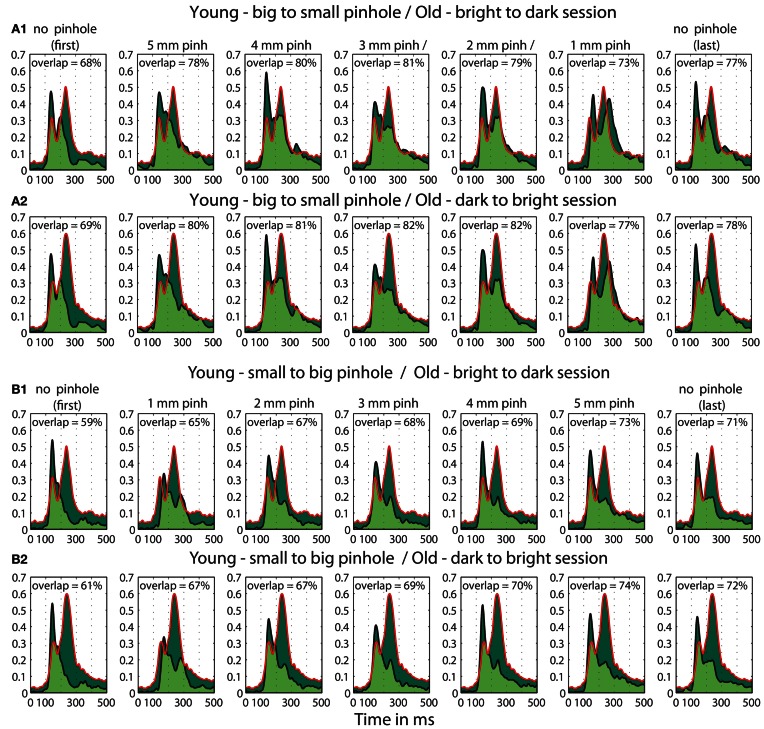
**Overlaps between *t*^2^ functions of young subjects tested in the pinhole experiment and old subjects tested in the luminance experiment. (A)** Overlap between old (>60) adults in the first brightest (60.8 cd/m^2^) condition from the b2d **(A1)** and d2b **(A2)** session of the luminance experiment and young subjects in all the pinhole conditions of the pinhole experiment's b2s session. **(B)** Overlap between old subjects in the brightest (60.8 cd/m^2^) condition from the b2d **(B1)** and d2b **(B2)** session of the luminance experiment and young subjects in all the pinhole conditions of the pinhole experiment's s2b session. Each subplot depicts the area under the *t*^2^ functions of young and old subjects, shaded in dark green. The edges of the *t*^2^ functions for young and old subjects are highlighted in black and in red, respectively. The overlap between *t*^2^ functions for young and old subjects is shaded in light green. The proportion of overlap is given inside each subplot.

Furthermore, we observed a qualitative difference in the shape of the *t*^2^ functions of young adults with no pinhole (and luminance level = 60.8 cd/m^2^) and old subjects tested at 60.8 cd/m^2^ (Figure 14). This shape difference was visible for all pinhole conditions. Additionally, in the 1 mm pinhole condition, the onsets of the face-texture ERP differences in young subjects were delayed compared to the onsets of the old adults in the luminance experiment (Figure 14). Our calculation of the overlap between *t*^2^ functions of young and old adults revealed that in both pinhole sessions, the overlap was 5–14% higher when young subjects wore pinholes compared to the no pinhole condition (Figure 15). For the b2s session the overlap was the highest for 2 and 3 mm pinholes ~81–82% (Figures 15A, **S2**), whereas for the s2b session it was the highest for the 5 mm pinhole ~73–74% (Figures 15B, **S3**). This result converges with our previous finding that 50ITs of young and old adults matched for 2 and 3 mm pinholes in b2s session and for the 5 mm pinhole in s2b session. Finally, the overlaps were 3–8% higher for the 5 mm pinhole size compared to 1 mm, which goes against our hypothesis that the smaller the pinhole in young subjects the bigger the overlap between young and old ERPs. Thus, although pinholes delay visual processing, they are not sufficient to make young subjects' ERPs look old.

## Discussion

Our study addressed several enduring questions about aging effects on visual processing speed, as reflected in ERPs. We measured by how much the visual processing of faces slows down throughout adulthood; the onset of the slowdown; and the contribution of luminance and pupil size to the age-related delay.

### Age-related ERP delays

First, our data confirms previous findings of Rousselet et al. (2010), suggesting that aging slows down visual processing speed by 1 ms/year. We extended this result by showing that this rate of slow-down is constant across luminance levels, from 60.8 to 0.59 cd/m^2^. At the highest luminance (60.8 cd/m^2^), the processing speed of older (>60) subjects was ~230 ms, about 50 ms slower than that of younger (<30) subjects (~180 ms). Aging also prolonged the latencies of maximum face-texture ERP differences, but at a sharper rate: on average by ~1.5 ms/year; the strongest face-texture ERP differences were observed ~140 ms post-stimulus in young adults, but only after 220 ms in older adults – about 80 ms later. Aging effects started around ~125 ms post-stimulus at 60.8 cd/m^2^ and, because of a main effect of luminance, were delayed up to 162 ms at 0.59 cd/m^2^; maximum aging effects appeared at ~200 ms at 60.8 cd/m^2^ and were delayed to ~260 ms at 0.59 cd/m^2^.

Previous research yielded inconclusive results regarding the dependency of aging effects on luminance: some research showed similar ERP aging delays regardless of the luminance level (Tobimatsu et al., 1993), whereas others suggested that ERP aging effects were stronger at low luminances (e.g., Shaw and Cant, 1980). The findings of Shaw and Cant (1980) do not contradict our observations—the luminance level beyond which their aging effect became weaker (~72 cd/ m^2^) is higher than our maximum luminance (60.8 cd/m^2^). Unfortunately, we are unable to relate the dependency between luminance and aging effects found in our study to other face ERP aging studies, because only one of them reported the mean luminance of their stimuli: 64 cd/m^2^ (Pfutze et al., 2002). In this study, Pfutze et al. (2002) reported no changes in P1 and N170 peak latencies with age. However, their results cannot be directly compared to our results because they did not consider the entire time-course of the effects, as is done in our approach. Nevertheless, it is possible that at luminances higher than the ones used in our study, ERP aging effects may decrease and future studies should address this question. Including information about luminance and contrast of the stimuli into method sections would also facilitate the comparison of age-related effects across studies.

In the current study, and the previous ones (Rousselet et al., 2009, 2010), our lab found ERP aging effects starting at about ~125 ms post-stimulus, and lasting for about 200 ms, with the strongest effects in the N170 time window. Aging effects in the N170 time window have been reported in several studies (Nakamura et al., 2001; Gazzaley et al., 2008; Wiese et al., 2008). However, we confirm that aging effects start earlier than the N170 peak, in keeping with the idea that peak analyses should be abandoned in favor of systematic time-point by time-point analyses (Rousselet and Pernet, 2011). Noteworthy, ERP studies using checkerboards have reported age-related latency increases already around 100–110 ms post-stimulus (Shaw and Cant, 1980; Sokol et al., 1981; Tobimatsu, 1995). However, checkerboards and faces differ in spatial frequency content, and it is thus difficult to compare the absolute latencies of the ERPs elicited by these two types of stimuli.

Among studies using faces (Chaby et al., 2001, 2003; Nakamura et al., 2001; Pfutze et al., 2002; Gazzaley et al., 2008; Wiese et al., 2008, 2012; Gao et al., 2009; Daniel and Bentin, 2010) discrepancies in N170 aging effects could be due to variability in stimulus parameters (e.g., whether or not external features were preserved, size, color, contrast, luminance), or ERP analysis approach (focused on peak amplitude and latency or peak-independent analyses), or both. It seems unlikely that particular stimuli could explain the presence of early aging effects because very different stimuli were used also among studies that did find delays in early ERPs, as well as among those that did not. Nevertheless, future work should determine how our aging effects are linked to potential differences in ERP information content, for instance using reverse correlation techniques (Schyns et al., 2009; Smith et al., 2012). This would allow us to determine if age-related changes in ERP shape are due to changes in diagnostic information, which might reflect, for instance, differences in task related strategies.

At present, it seems more plausible that the differences in ERP aging effects between our study and the existing literature stem from the application of different measures of age-related delays. Most studies focus on component peak latencies in pre-defined time windows, whereas our analyses take into consideration changes in the overall shape of the ERP, and is independent of ERP peaks and regions of interests. Thus, it is entirely possible that similar aging effects would be obtained by applying our approach to data from other studies. Moreover, the 1 ms/year age-related delay in processing speed, obtained with our analysis approach, has been replicated both within studies (testing our subjects twice), and across studies in independent samples of subjects from two countries (Rousselet et al., 2009, 2010).

### Luminance effect on the ERPs

We found that, independently of age, luminance strongly modulated ERPs. Previous research has shown that decreasing luminance increases the latencies of neuronal responses in cortical areas including V1 (Geisler et al., 2007), the superior colliculus (Marino et al., 2012) and the LIP - lateral intraparietal area (Tanaka et al., 2013). Early ERPs (~100 ms) are also delayed by changes in luminance (Wicke et al., 1964; Cant et al., 1978; Tobimatsu et al., 1993; Johannes et al., 1995). Our study extends this finding by showing that luminance affects most of the ERP time-course, within 500 ms post-stimulus, starting about 60 ms post-stimulus, with maximum modulations occurring between 130 and 150 ms. These strongest luminance effects occurred after the P100 time-window, a period of activity commonly thought to be most sensitive to changes in low-level visual factors, such as luminance (Shaw and Cant, 1980), contrast (MacKay and Jeffreys, 1973), size (Yiannikas and Walsh, 1983) or color (Anllo-Vento and Hillyard, 1996). Our results suggest that it is not the P100 but the 130–150 ms period that is most strongly modulated by luminance—the period usually associated with higher-order cognitive processes, such as object and face categorization (Itier and Taylor, 2004), expertise (Tanaka and Curran, 2001), or task-related processes (Rousselet et al., 2011). Stronger sensitivity to changes in luminance around 130–200 ms, rather than around 100 ms, has also been observed in a study using short flashes of vertical bars (Johannes et al., 1995). Thus, visual ERP studies should not underestimate the effects of low-level factors beyond the P1 time window. Reporting the screen luminance is also essential to be able to compare ERP latencies across studies.

### Contribution of pupil size and senile miosis to age-related ERP delays

Our data show that pupil size decreases with aging at the rate of ~0.03 mm/year at 60.8 cd/m^2^, ~0.04 mm/year at intermediate luminances, and ~0.05 mm/year at 0.59 cd/m^2^. This is equivalent to about 0.6–1 mm reduction every 20 years. These estimates match quite well those obtained in previous studies—for instance Winn et al. (1994) found a decrease in pupil size of about 0.03 mm/year at 220 cd/m^2^ and ~0.04 mm/year at 44 and 9 cd/m^2^. Birren et al. (1950) reported a 2.5 mm difference in pupil size between subjects in their twenties and subjects in their eighties (~0.04 mm/year) at 3.18 cd/m^2^. Between the same age groups, Sokol et al. (1981) observed a slightly smaller reduction in pupil size of 1.5 mm (0.025 mm/year) at 1.9 cd/m^2^. Finally, our senile miosis measurements fit well with a recent model that estimates pupil size based on age, luminance, size of adaptive field and whether one or two eyes have been adapted (Watson and Yellott, 2012).

Contrary to our expectations, senile miosis is unlikely to be a factor explaining age-related delays in visual ERPs. Moreover, individual variability in pupil size within age groups cannot account for individual differences in visual processing speed. First, after partialling out the effect of age from our processing speed measurements and from pupil size, we failed to find a relationship between processing speed and pupil size. Second, at 31 and 16 cd/m^2^, the luminance conditions providing the best retinal illuminance match between young and old subjects, the overlap between their ERPs was the second smallest (after 60.8 cd/m^2^). Additionally, in the conditions where young-old ERP overlap was the highest (0.59, 1.14, 2.17 cd/m^2^), the retinal illuminance of young subjects was only about 5–10% of that of old adults. Furthermore, in experiment 2, we failed to match the ERPs of old subjects tested at high luminance to those of young subjects wearing pinholes. In fact, we found a counterintuitive result: the young-old ERP overlap was 3–8% higher in the 5 mm pinhole size condition compared to the 1 mm condition. Overall, our results demonstrate that ERPs to faces are delayed by aging at the early stages of visual processing (< 200 ms) and strongly suggest that these delays are of cortical, rather than optical origin.

### Contribution of other optical factors and contrast sensitivity to ERP aging delays

Ruling out senile miosis as possible contributor to age-related visual processing delays does not necessarily mean that no other optical factors are involved. With age, there is a reduction in lens light transmittance (Boettner and Wolter, 1960), as well as in the eye's ability to accommodate, which decreases from the fifth decade of life onwards and seems to disappear altogether in the sixth decade (Birren and Schaie, 2001). Additionally, after the age of 40, the amount of intraocular light scatter increases, leading to a reduction in retinal image contrast (Fujisawa and Sasaki, 1995). Under these circumstances, senile miosis is actually beneficial because it diminishes optical aberrations (Applegate et al., 2007); it also boosts depth of focus, improving contrast and the overall quality of the retinal image (Weale, 1992). Despite the positive effects of senile miosis, overall, reduction in pupil diameter, increase scatter and a decrease in ocular transmittance lead to ~60% of light loss at the retina, at lower light levels, between the ages of 20 and 60 years. However, this reduced retinal illuminance in old subjects is unlikely to account for the ERP aging delays found in our study because even when retinal illuminances of young and old subjects matched, their ERPs did not.

Another factor that could potentially contribute to our ERP aging delays is a decline in spatial contrast sensitivity with age. The effects of diminished contrast sensitivity in the elderly have been primarily studied at the early stages of visual processing, especially around the P100 (Morrison and Reilly, 1989; Tobimatsu et al., 1993; Tobimatsu, 1995). Morrison and Reilly (1989) showed that incrementing stimulus contrast makes ERPs of older observers resemble those of young observers. Tobimatsu et al. (1993) found that a reduction in contrast of checkerboard patterns leads to significant differences in P100 latency between young and middle age groups, contrary to high contrast checks for which no difference was observed. In the older group, P100 latencies were delayed compared to the middle age group for both low and high contrast checks. Our stimuli had RMS contrast of 0.1, which is similar to the low contrast stimuli used by Tobimatsu et al. (1993). It is therefore possible that for higher contrast stimuli, our aging effects would be less pronounced; to our knowledge no face ERP study has yet addressed the link between stimulus contrast and aging delays.

However, it is unlikely that reduction in contrast sensitivity could fully explain our ERP aging delays. Contrast sensitivity loss under photopic light conditions has been observed in particular for intermediate and high spatial frequencies—above 2 cycles/degree of visual angle (Owsley et al., 1983). In our stimuli, 90% of the total power was contained within the low to intermediate spatial frequency range (Bieniek et al., 2012, Figure 1)—below 20 cycles/image, which for our image size of 9° of visual angle is equivalent to ~2.2 cycles/degree. This suggests that most of the spatial frequency content of our images is below the range that is typically affected by aging. Also, age-related differences in contrast sensitivity are larger under mesopic and scotopic light conditions than in photopic conditions (Sloane et al., 1988; Owsley, 2011). In our study, only the 0.59 cd/m^2^ luminance condition falls within the mesopic range. However, the aging effect for that luminance level did not differ from the one observed at the highest luminance level. Thus, a link between our aging effects and contrast sensitivity loss is unlikely.

### Possible accounts of the ERP aging effects

Finally, in this last section, we speculate about the main factors that could account for age-related processing speed slow-down, including: alteration in axons' myelination, reduced synaptic and network efficiency, decrease in neuronal response selectivity, inhibitory deficits, and neuronal network reorganization.

First, slow-down of visual processing speed with age may be due to myelin alteration: aging is associated with degeneration of myelin sheaths of cortical neurons that subsequently get remyelinated, but with shorter internodes, leading to slower conduction along nerve fibers (Peters, 2002, 2009). Changes in myelin sheaths are distributed across gray matter and white matter, suggesting that communication both within and between cortical regions might be disturbed (Peters, 2002). In keeping with these anatomical observations, there is direct evidence for age-related slowing in the visual system: Wang et al. (2005) reported delays in the latency of inter-cortical spiking activity, between V1 and V2, as well as intra-cortical activity, within V1 and V2, and this effect was more pronounced in V2 compared to V1.

In humans, post-mortem analysis reveals stronger changes in white-matter density than in gray-matter density with healthy aging (Piguet et al., 2009). Using *in vivo* techniques, several studies have suggested a relationship between age-related decline in white matter and cognitive function including speed of processing (Bucur et al., 2008; Eckert et al., 2010; Eckert, 2011; Salami et al., 2012). However, some of these studies potentially suffer from a statistical problem arising from the artificial correlation between time-dependent variables (Hofer and Sliwinski, 2001; Lazic, 2010). These studies also use composite behavioral measures of processing speed that do not have the specificity and the temporal resolution potentially afforded by EEG and MEG.

Additionally, animal studies suggest that aging is associated with a decrease in spine numbers and spine density (Duan et al., 2003), as well as with alterations in the strength and efficiency of synaptic connections (Mostany et al., 2013). Although at a different scale, reduced efficiency of cortical networks in older individuals has also been suggested in humans (Achard and Bullmore, 2007). This loss in efficiency may be linked to the degradation of neuronal response selectivity, which in turn could translates into slower processing times. Indeed, animal research shows that aging is associated with an increase in spontaneous activity of neurons, a reduction in signal to noise ratio, and a deterioration of orientation and direction selectivity in V1 and V2 (Schmolesky et al., 2000; Hua et al., 2006; Yu et al., 2006). This increase in noise and decrease in selectivity of neuronal responses may lead to broader tuning of neuronal populations and impair face-specialized processing. This notion is supported by fMRI findings of reduced differentiation of BOLD signal between faces and pink noise textures (Park et al., 2004) accompanied by an increase in BOLD response to all categories in regions normally preferentially active for certain categories only (Park et al., 2004; Payer et al., 2006; Voss et al., 2008). If populations of cells become less tuned to a specific stimulus, the rate of accumulation of evidence supporting its recognition would slow down, leading to longer processing times (Perrett and Ashbridge, 1998).

The deterioration of visually driven neuronal responses has also been linked to an age-related reduction in GABA concentration. The administration of GABA to V1 cells of senescent monkeys' improved selectivity of visual responses, demonstrating a direct link between inhibitory processes and healthy visual function (Leventhal et al., 2003). In humans, inhibitory deficits in elderly subjects have been captured at the level of populations of neurons using EEG. For instance, Gazzaley et al. (2008) found that older adults have more difficulties with suppressing task-irrelevant information, which manifests itself in longer N170 latencies (but unaffected P1 latencies). It is unclear whether our results of most prominent aging effects occurring in the N170 time window can be linked to inhibitory deficits or decreased specialization of face-selective processes: further research should address this question.

Although they cannot yet be linked to particular processes, it seems plausible that our earliest aging effects (~125) involve activity from higher-order visual areas. Intracranial recordings showed face-sensitive responses in extrastriate areas (Halgren et al., 1994), occipital and temporal structures (Liu et al., 2009), and in the fusiform gyrus (Barbeau et al., 2008) as early as ~100 ms. Strikingly, one small cortical patch can generate the whole P1-N170-P2 complex (Allison et al., 1999; Sehatpour et al., 2008; Rosburg et al., 2010). Studies using scalp recordings have also reported responses differentiating between faces and other objects already ~100 ms post stimulus (Linkenkaer-Hansen et al., 1998; Pizzagalli et al., 1999; Yamamoto and Kashikura, 1999; Halit et al., 2000; Itier and Taylor, 2002; Liu et al., 2002; Herrmann et al., 2005). However, some of these studies used non-causal filters with relatively large high-pass cut-offs between 0.8 and 1.5 Hz, which could have shortened onsets by smearing effects back in time (Acunzo et al., 2012; Rousselet, 2012; Widman and Schroeger, 2012). In our study we used a causal Butterworth high-pass filter, which does not distort onsets, and found face-texture ERP differences starting around 90 ms post-stimulus. This suggests that the visual system detects faces very rapidly, and that aging starts to affect visual processes within 35–40 ms after face detection.

However, if degeneration of myelin and increased noise of neuronal responses are visible already in V1 (Schmolesky et al., 2000; Peters et al., 2001), we would expect to see ERP aging differences earlier than ~125 ms post-stimulus. This is assuming serial processing from V1 onward, and our capacity to measure evoked responses from all successive stages, which is a rather unrealistic model (Foxe and Simpson, 2002). Additionally, face stimuli are not optimal to capture very early brain activity, and different strategies have been suggested to measure the earliest cortical onsets, as reflected in the C1 component, starting around 60 ms post-stimulus (Kelly et al., 2008). Whether age-related differences in activity from striate and early extra-striate areas might occur in the absence of differences in the onset of face related areas remain to be investigated.

Reduced selectivity of neuronal responses and deficits in inhibition of irrelevant information may lead to slower accumulation of evidence useful for decision making. It has been suggested that subjects' behavioral choices can be predicted from the activity in two EEG time windows associated with the accumulation of evidence useful for decision making: one early (~N170) and one late (>300 ms) (Philiastides and Sajda, 2006; Philiastides et al., 2006). In our study, aging effects started 35–40 ms after the onsets of face/texture differences. Moreover, subjects' behavioral performance did not change with age and was close to 100%. Thus, it seems plausible that, for all age groups, stimulus processing starts at the same time, but when the aging effects appear, the whole cascade of information accumulation necessary for a behavioral decision is disturbed, leading to longer processing times, without necessarily hampering subjects' performance—at least in an easy task such as ours. The task used in our study was designed to be very easy in order to measure age-related ERP differences in processing speed in the absence of behavioral differences.

Age-related neuronal changes might also lead to the involvement of additional or different neuronal circuits—reorganizations that could potentially explain our aging results. Indeed, age-related reorganization of neuronal networks during face processing has been observed by Grady et al. (2000). They discovered that in young adults better recognition of degraded face images was positively correlated with the activity in the fusiform gyrus, in contrast to old adults for whom behavioral performance correlated with activity in the posterior occipital cortex. Other studies found that when task difficulty increases (for instance because faces are degraded), older observers rely more on prefrontal areas, suggesting that, with age, there is an over recruitment of frontal activity to compensate for poorer performance of the sensory systems (Grady, 2008). In our study, the behavioral task was very simple, most likely not requiring the involvement of compensatory brain circuits. However, the exact task conditions that promote frontal compensation in old adults are still poorly understood. Also, evidence for over-recruitment and compensation have been obtained from cross-sectional designs, and have been challenged by a recent longitudinal study (Nyberg et al., 2010, 2012).

Finally, our aging effects could be related to a decline in perceptual grouping abilities (Kurylo, 2006) and contour integration (Roudaia et al., 2008, 2011). Because face and object recognition rely to a large extent on contours and edges carried by image phase information (Gaspar and Rousselet, 2009; Bieniek et al., 2012), any deficit in a mechanism responsible for contour integration is likely to affect face and object processing. An important research question would thus be to determine the relationship between ERP aging delays and age-related contour integration deficits, which might themselves be due to inhibitory deficits and other neuronal changes.

### Conflict of interest statement

The authors declare that the research was conducted in the absence of any commercial or financial relationships that could be construed as a potential conflict of interest.
