# Supplementary material for: Early ERPs to faces: aging, luminance, and individual differences
Source: Front Psychol. 2013 May 14;4:268. doi: 10.3389/fpsyg.2013.00268 (PMC3653118; doi:10.3389/fpsyg.2013.00268)
Supplement: Supplementary file 1 [file Presentation1.PDF]

Early ERPs to faces: aging, luminance and individual differences

Magdalena M. Bieniek[1*], Luisa Frei[1,2], Guillaume A. Rousselet[1]

(1) Institute of Neuroscience and Psychology, College of Medical, Veterinary and Life Sciences, University of Glasgow, 58 Hillhead Street, G12 8QB, Glasgow, UK.

(2) Sackler Institute of Psychobiological Research, Institute of Health and Wellbeing College of Medical, Veterinary and Life Science, University of Glasgow, Neurosurgery Building Southern General Hospital, G51 4TF, Glasgow UK.

*** Corresponding author:**

Address: Institute of Neuroscience and Psychology, 58 Hillhead Street, G12 8QB, Glasgow, UK.

Telephone: 00 44 (0) 141 330 3610

Email: Magdalena.Bieniek@glasgow.ac.uk

Supplementary Material

Supplementary Table 1. Age regression fits in the b2d session. Confidence intervals around the slopes and intercepts (intr) are given in square brackets.

	Pupil size		Retinal illuminance		Onset		Amplitude		50IT		Peak latency	
lum	slope	intr	slope	intr	slope	intr	slope	intr	slope	intr	slope	intr
60.8 (first)	-0.03 [-0.04, -0.02]	5.93 [5.47, 6.41]	-12.48 [-17.82, -7.33]	1471 [1163.5, 1791.4]	0.06 [-0.24, 0.43]	95.8 [81.83, 110.84]	-0.01 [-0.06, 0.06]	13.38 [10.20, 16.68]	1.05 [0.75, 1.33]	160.01 [144.5, 175.12]	1.38 [0.63, 2.09]	106.8 [84.31, 130.81]
31	-0.04 [-0.05, -0.03]	6.67 [6.16, 7.21]	-9.01 [-12.81, -5.87]	964.43 [766.44, 1207.8]	0.3 [-0.01, 0.67]	86.70 [71.13, 102.22]	-0.01 [-0.09, 0.05]	13.92 [10.91, 17.55]	0.96 [0.73, 1.23]	172.48 [158.58, 184.03]	1.33 [0.73, 2.07]	119.87 [94, 142.17]
16	-0.04 [-0.05, -0.03]	7.11 [6.71 ,7.56]	-5.52 [-7.2, -3.61]	575.06 [459.26, 678.15]	0.21 [-0.17, 0.64]	92.02 [74.97, 110.84]	-0.01 [-0.07, 0.06]	13.19 [10.09, 16.7]	1.05 [0.77, 1.36]	173.68 [155.64, 89.57]	1.32 [0.73, 2.17]	127.62 [98, 149.36]
8.16	-0.04 [-0.05, -0.03]	7.37 [6.95, 7.82]	-2.78 [-3.74, -1.74]	299.10 [236.2, 355.64]	0.029 [-0.21, 0.33]	104.90 [92.13, 116.43]	0.01 [-0.05, 0.07]	12.01 [8.62, 15.21]	1.00 [0.78, 1.22]	181.41 [170.71, 195.97]	1.73 [0.87, 2.26]	117.04 [97.11, 144.95]
4.19	-0.04 [-0.05 ,-0.03]	7.55 [7.11, 8.01]	-1.50 [-2.01, -1.00]	162.65 [131.0, 192.92]	0.10 [-0.11, 0.53]	106.28 [92.36, 115.79]	0.01 [-0.06, 0.08]	11.77 [8.1, 15.66]	1.01 [0.72, 1.34]	190.81 [174.12, 207.35]	1.72 [1.03, 2.23]	129.94 [106.25, 158.95]
2.17	-0.04 -0.05, -0.03]	7.62 [7.13, 8.09]	-0.75 [-1.07, -0.46]	84.55 [66.55, 103.47]	0.06 [-0.15, 0.78]	112.32 [92.48, 123.97]	-0.01 [-0.07, 0.05]	12.61 [9.49, 16.19]	1.02 [0.79, 1.26]	196.18 [184.14, 207.78]	1.5 [0.9, 2.11]	150.78 [123.3, 175.95]
1.12	-0.04 [-0.05, -0.03]	7.73 [7.25, 8.21]	-0.39 [-0.57, -0.25]	44.43 [35.07, 54.17]	0.38 [0.12, 0.7]	108.15 [93.2, 119.59]	0.01 [-0.05, 0.07]	11.49 [8.50, 14.74]	1.04 [0.75, 1.34]	203.78 [189.14, 219.85]	1.8 [1.3, 2.27]	147.62 [121.09, 174.69]
0.59	-0.04 [-0.06, -0.04]	7.81 [7.34, 8.27]	-0.21 [-0.31, -0.18]	24.43 [19.66, 29.53]	0.62 [0.31, 1.38]	105.29 [85.09, 121.35]	0.005 [-0.05, 0.06]	10.92 [8.41, 14.39]	0.94 [0.72, 1.19]	219.81 [208.37, 231.00]	1.10 [0.4, 1.85]	186.62 [149.6, 221.88]
60.8 (last)	-0.03 [-0.04, -0.02]	5.43 [4.85, 5.94]	-9.49 -[13.98, -5.047]	1184 [919.68, 1463]	0.08 [-0.18, 0.41]	91.56 [75.68, 105.43]	-0.01 [-0.07, 0.05]	13.64 [10.47, 17.16]	0.75 [0.36, 1.18]	181.17 [158.96, 203.35]	1.36 [0.72, 2.06]	115.81 [89.37, 140.48]

Supplementary Table 2. Age regression fits in the d2b session. Confidence intervals of the slopes and intercepts (intr) are given in square brackets.

	Pupil size		Retinal illuminance		Onset		Amplitude		50IT		Peak latency	
lum	slope	intr	slope	intr	slope	intr	slope	intr	slope	intr	slope	intr
60.8 (first)	-0.03 [-0.04, -0.02]	5.95 [5.36, 6.52]	-11.15 [-15.72, -6.47]	1433.51 [1175.8, 1699.7]	0.27 [0.03, 0.55]	82.6 [69.27, 95.015]	0 [-0.06, 0.06]	13.31 [10.81, 16.04]	1.07 [0.64, 1.38]	158.71 [143.04, 177.20]	1.27 [0.61, 2.05]	116.86 [85.84, 140.73]
0.59	-0.05 [-0.06, -0.03]	8.11 [7.65, 8.55]	-0.24 [-0.30, -0.15]	27.07 [22.08, 30.32]	0.40 [0.13, 0.73]	115 [102.03, 127.27]	0 [-0.05, 0.071]	11.4 [8.50, 13.87]	1.22 [0.92, 1.5]	201.04 [186.37, 216.25]	1.59 [0.90, 2.39]	159.05 [117.92, 199.8]
1.12	-0.05 [-0.05, -0.03]	7.79 [7.30, 8.23]	-0.40 [-0.49, -0.27]	45.95 [38.65, 51.02]	0.36 [0.07, 0.64]	112.25 [100.67, 124.71]	0.01 [-0.03, 0.073]	11.63 [8.92, 13.98]	1.07 [0.75, 1.37]	202.72 [186.12, 219.639]	1.52 [0.73, 2.26]	160.34 [121.43, 202.8]
2.17	-0.05 [-0.05, -0.03]	7.61 [7.07, 8.12]	-0.79 [-0.97, -0.52]	87.66 [71.65, 98.50]	0.48 [0.18, 1.09]	95.12 [76.86, 108.51]	0.01 [-0.05, 0.07]	11.54 [8.54, 14.442]	1.01 [0.68, 1.35]	197.07 [179.46, 215.15]	1.76 [1.09, 2.35]	136.12 [104.71, 166.55]
4.19	-0.04 [-0.05, -0.03]	7.43 [6.93, 7.84]	-1.38 [-1.72, -0.94]	156.50 [130.49, 176.04]	0.18 [-0.10, 0.72]	98.38 [82.77, 115.94]	0.01 [-0.05, 0.079]	11.99 [9.29, 14.50]	0.95 [0.64, 1.22]	193.34 [177.53, 211.16]	1.78 [1.28, 2.25]	130 [104.17, 153.75]
8.16	-0.04 [-0.05, -0.03]	7.03 [6.59, 7.46]	-2.35 [-2.96, -1.52]	268.91 [225.32, 304.02]	0.29 [0.05, 0.58]	89.68 [77.68, 101.03]	0.01 [-0.04, 0.07]	11.96 [9.14, 14.67]	0.91 [0.54, 1.27]	189.41 [171.03, 208.82]	1.48 [0.9, 2.07]	135.69 [108.40, 159.63]
16	-0.04 [-0.05, -0.03]	6.7 [6.23, 7.17]	-4.01 [-5.15, -2.70]	476.12 [405.92, 548.24]	0.11 [-0.18, 0.44]	94.4 [79.92, 108.07]	0.01 [-0.04, 0.08]	12.26 [9.41, 15]	1.03 [0.68, 1.338]	173.77 [157.81, 193.44]	1.42 [0.78, 2.17]	123.24 [91.80, 147.64]
31	-0.04 [-0.04, -0.02]	6.27 [5.74, 6.71]	-6.57 [-8.83, -4.203]	813.75 [686.97, 952.64]	0.14 [-0.17, 0.49]	90.43 [74.02, 105.44]	0.02 [-0.04, 0.08]	12.32 [9.39, 15.16]	0.90 [0.55, 1.23]	174.92 [157.12, 194.47]	1.17 [0.55, 1.87]	130.69 [99.69, 156.913]
60.8 (last)	-0.03 [-0.04, -0.02]	5.74 [5.21, 6.19]	-10.62 [-14.42, -6.55]	1335.97 [1104.7, 1552.8]	0.16 [-0.12, 0.44]	86.33 [73.8, 98.53]	0.01 [-0.04, 0.07]	12.37 [9.70, 15.62]	0.97 [0.68, 1.194]	168.77 [156.02, 183.20]	1.20 [0.66, 1.88]	124.06 [98.03, 144.64]

Supplementary Table 3. Age regression slopes and intercepts differences between the first brightest luminance and all the other luminance conditions in the b2d session.

	**Pupil size**		**Retinal illuminance**		**Onset**		**Amplitude**		**50IT**		**Peak latency**	
**lum**	**slope**	**intr**	**slope**	**intr**	**slope**	**intr**	**slope**	**intr**	**slope**	**intr**	**slope**	**intr**
**31**	0.01 [0, 0.01]	-0.76 [-0.98, -0.568]	-3.46 [-5.978, -0.77]	506 [341, 646]	-0.24 [-0.54, 0.04]	9.09 [-2.81, 22.93]	0.01 [-0.02, 0.04]	-0.54 [-2.02, 0.80]	0.09 [-0.13, 0.31]	-12.48 [-24, -2]	0.04 [-0.6, 0.45]	-13.08 [-26.95, 4.91]
**16**	0.01 [0, 0.02]	-1.18 [-1.42, -0.928	-6.96 [-11.13, -3.13]	896 [674, 1144]	-0.15 [-0.44, 0.14]	3.77 [-11.34, 19.8]	0 [-0.02, 0.03]	0.2 [-1.14, 1.48]	0 [-0.2, 0.18]	-13.67 [-22.17, -4.25]	0.06 [-0.76, 0.66]	-20.83 [-42.03, 2.45]
**8.16**	0.01 [0, 0.02]	-1.44 [-1.68, -1.17	-9.69 [-14.52, -5.24]	1172 [906, 1450]	0.03 [-0.27, 0.31]	-9.11 [-20.51, 4.16]	-0.02 [-0.05, 0.01]	1.38 [0.15, 2.76]	0.04 [-0.15, 0.24]	-21.41 [-32.41, -11.20]	-0.36 [-0.90, 0.15]	-10.24 [-30.94, 6.85]
**4.19**	0.01 [0, 0.02]	-1.62 [-1.91, -1.3	-10.97 [-16.23, -6.25]	1308 [1013, 1617]	-0.04 [-0.43, 0.21]	-10.48 [-20.24, 3.21]	-0.02 [-0.06, 0.02]	1.62 [-0.5, 4.02]	0.03 [-0.22, 0.3]	-30.81 [-45.70, -15.94]	-0.34 [-0.95, 0.26]	-23.14 [-51.4, -0.59]
**2.17**	0.01 [0, 0.02]	-1.69 [-1.94, -1.40	-11.72 [-17.11, -6.92]	1386 [1090, 1704]	0 [-0.59, 0.20]	-16.53 [-25.61, 1.74]	0.01 [-0.03, 0.04]	0.77 [-1.30, 2.94]	0.03 [-0.2, 0.25]	-36.18 [-47.32, -24.50]	-0.12 [-0.89, 0.53]	-43.99 [-73.02, -15.72]
**1.12**	0.01 [0, 0.02]	-1.8 [-2.05, -1.51	-12.08 [-17.64, -7.13]	1426 [1122, 1753]	-0.31 [-0.7, 0.1]	-12.36 [-28.21, 3.93]	-0.02 [-0.06, 0.04]	1.9 [-0.49, 4.4]	0 [-0.29, 0.26]	-43.78 [-57.68, -28.7]	-0.42 [-1.18, 0.27]	-40.82 [-71.2, -11.15]
**0.59**	0.01 [0, 0.02]	-1.88 [-2.17, -1.6]	-12.26 [-17.85, -7.22]	1446 [1136, 1776]	-0.56 [-1.20, -0.16]	-9.49 [-27.28, 10.26]	-0.01 [-0.059, 0.04]	2.46 [0.27, 4.74]	0.11 [-0.2, 0.39]	-59.81 [-74.10, -44.38]	0.28 [-0.52, 0.97]	-79.82 [-113.6, -47.54]
**60.8 (last)**	0 [-0.01, 0]	0.5 [0.19, 0.85]	-2.98 [-6.69, -0.04]	286.37 [112.1, 522.70]	-0.02 [-0.28, 0.27]	4.23 [-7.73, 16.74]	0.01 [-0.019, 0.032]	-0.25 [-1.36, 1.10]	0.3 [0.05, 0.54]	-21.17 [-34.07, -6.89]	0.02 [-0.57, 0.53]	-9.01 [-26.44, 10.35]

Supplementary Table 4. Age regression slopes and intercepts differences between the first brightest luminance and all the other luminance conditions in the d2b session.

	**Pupil size**		**Retinal illuminance**		**Onset**		**Amplitude**		**50IT**		**Peak latency**	
**lum**	**slope**	**intr**	**slope**	**intr**	**slope**	**intr**	**slope**	**intr**	**slope**	**intr**	**slope**	**intr**
**0.59**	0.02 [0,0.02]	-2.16 [-2.61, -1.66]	-10.91 [-15.46, -6.4]	1406 [1171, 1659]	-0.13 [-0.47, 0.14]	-32.38 [-45.87, -19.18]	0 [-0.04, 0.04]	1.92 [0.10, 3.69]	-0.15 [-0.54, 0.16]	-42.34 [-59.82, -23.97]	-0.31 [-1.1, 0.40]	-42.19 [-85.6, -2.77]
**1.12**	0.01 [0,0.02]	-1.83 [-2.26, -1.4]	-10.75 [-15.28, -6.22]	1387 [1152, 1639]	-0.09 [-0.37, 0.23]	-29.66 [-43.89 -17.78]	-0.01 [-0.05, 0.03]	1.69 [0.08, 3.42]	0 [-0.33, 0.3]	-44.01 [-60, -27.41]	-0.24 [-0.82, 0.42]	-43.48 [-82.4, -14.58]
**2.17**	0.01 [0,0.02]	-1.66 [-2.05, -1.20]	-10.36 [-14.81, -5.87]	1345 [1118, 1596]	-0.21 [-0.72, 0.04]	-12.53 [-25.25, 6.77]	-0.01 [-0.03, 0.02]	1.78 [0.42, 3.14]	0.05 [-0.29, 0.36]	-38.36 [-54.12, -20.52]	-0.48 [-1.13, 0.16]	-19.26 [-50.8, 9.19]
**4.19**	0.01 [0,0.02]	-1.48 [-1.88, -1.05]	-9.77 [-14.06, -5.36]	1277 [1051, 1518]	0.09 [-0.35, 0.41]	-15.78 [-33.14, -2.37]	-0.01 [-0.04, 0.03]	1.33 [0.02, 2.8]	0.11 [-0.16, 0.38]	-34.63 [-48.41, -22.76]	-0.51 [-1.07, 0.11]	-13.14 [-37.6, 5.95]
**8.16**	0.01 [0,0.02]	-1.08 [-1.44, -0.72]	-8.8 [-12.80, -4.72]	1164 [954, 1398]	-0.02 [-0.28, 0.24]	-7.08 [-20.63, 7.01]	-0.01 [-0.04, 0.02]	1.36 [0.04, 2.71]	0.15 [-0.13, 0.46]	-30.7 [-46.89, -17.54]	-0.21 [-0.69, 0.29]	-18.82 [-42.5, 0.04]
**16**	0.01 [0,0.01]	-0.75 [-1.11, -0.37]	-7.14 [-11.01, -3.42]	957 [765, 1178]	0.16 [-0.14, 0.48]	-11.8 [-26.59, 2.97]	-0.01 [-0.05, 0.02]	1.06 [-0.27, 2.48]	0.04 [-0.22, 0.28]	-15.06 [-28.24, -1.70]	-0.15 [-0.72, 0.34]	-6.38 [-23.8, 13.14]
**31**	0 [0,0.01]	-0.32 [-0.67, 0.045]	-4.58 [-7.85, -1.38]	619 [459, 812]	0.13 [-0.16, 0.41]	-7.83 [-20.80, 6.54]	-0.02 [-0.05, 0.01]	0.99 [-0.33, 2.52]	0.16 [-0.16, 0.51]	-16.21 [-35.32, 1.38]	0.11 [-0.32, 0.62]	-13.83 [-36.1, 4.17]
**60.8 (last)**	0 [0,0]	0.22 [-0.11, 0.55]	-0.53 [-3.24, 2.30]	97 [-61, 264]	0.11 [-0.15, 0.39]	-3.74 [-16.43, 9.28]	-0.01 [-0.05, 0.02]	0.95 [-0.39, 2.24]	0.1 [-0.21, 0.36]	-10.06 [-24.90, 6.02]	0.07 [-0.27, 0.38]	-7.2 [-18.9, 3.72]

Supplementary Table 5. Slopes and intercepts of regressions of 50IT and peak latency against pupil size, after partialling out the effects of age. Confidence intervals of the slopes and intercepts are given in square brackets.

	**B2D**				**D2B**			
	**50IT / pupil size**		**Peak lat / pupil size**		**50IT / pupil size**		**Peak lat / pupil size**	
**luminance**	slope	intercept	slope	intercept	slope	intercept	slope	intercept
**60.8 (first)**	-1.67 [-8.68, 7.47]	-0.08 [-6.22, 5.34]	1.7 [-12.44, 18.7]	0.02 [-9.72, 10.62]	-6.19 [-15.19, 6.08]	-0.49 [-7.45, 7.27]	0.50 [-29, 0.44]	-13.73 [-10.113, 13.31]
**31**	1.73 [-3.89, 7.85]	0.03 [-4.84, 4.49]	3.05 [-11.49, 17.98]	0.27 [-9.85, 11.87]	-3.18 [-8.62, 3.89]	0.14 [-4.50, 5.29]	-0.21 [-21.43, 12.42]	-6.86 [-11.93, 11.26]
**16**	-1.66 [-8.73, 7.90]	-0.04 [-5.20, 5.29]	-7.92 [-23.02, 10.73]	-0.53 [-12.20, 10.6]	-5.56 [-11.82, 1.78]	0.23 [-4.97, 5.71]	0.14 [-20.09, 10.14]	-7.38 [-10.66, 10.81]
**8.16**	-0.22 [-7.43, 8.68]	0.06 [-4.58, 5.03]	8.36 [-12.92, 26.35]	-0.4 [-13.02, 12]	-5.83 [-11.73, 0.65]	0.11 [-5.03, 6.07]	-0.16 [-22.50, 4.33]	-9.71 [-10.53, 9.55]
**4.19**	1.03 [-6.67, 8.88]	0.06 [-5.88, 6.37]	-8.31 [-25.88, 12.85]	-0.21 [-10.85, 9.94]	-1.91 [-9.46, 7.59]	-0.31 [-5.90, 5.88]	0.02 [-25.42, 10.44]	-5.6 [-10.56, 10.62]
**2.17**	-1.04 [-8.03, 5.57]	0.03 [-4.58, 4.51]	-15.9 [-27.21, -2.46]	-0.41 [-12.35, 10.29]	0.12 [-7.54, 8.23]	0.03 [-6.13, 6.40]	-0.63 [-20.89, 6.95]	-9.48 [-11.14, 10.59]
**1.12**	-2.36 [-9.17, 5.74]	-0.003 [-5.48, 5.19]	-13.21 -24.65, -1.54]	-0.43 [-10.16, 9.72]	-1.32 [-8.06, 6.13]	0.09 [-6.64, 6.39]	-0.04 [-13.67, 14.18]	-1.71 [-13.37, 17.43]
**0.59**	-5.11 [-12.90, 1.85]	0.33 [-3.89, 5.54]	-16.06 -30.34, 2.54]	0.21 [-13.33, 15.37]	-2.11 [-9.19, 8.29]	-0.10 [-5.55, 5.64]	-0.13 [-15.4, 13.20]	0.15 [-11.81, 14.95]
**60.8 (last)**	-4.72 [-14.67, 5.12]	0.005 [-6.99, 6.37]	-12.75 -26.72, 3.73]	0.02 [-10.34, 9.97]	-1.95 [-8.89, 5.45]	-0.05 [-4.52, 5.25]	0.06 [-20.41 10.87]	-4.8 [-9.52, 10.96]

Supplementary Table 6. 50IT and peak latency differences (ms), between young (<30) and old (>60) subjects. Differences in median processing speed (50IT) and median peak latency of face-texture ERP difference between young subjects in all luminance conditions, and old subjects in the first brightest condition (luminance = 60.8 cd/m^2). For each difference the 95% bootstrap confidence interval is given in square brackets.

B2d sessions			D2b sessions		
**luminance**	**50IT**	**Peak latency**	**luminance**	**50IT**	**Peak latency**
**60.8 (first)**	-50 [-64, -34]	-84 [-100, -33]	**60.8 (first)**	-53 [-72, -33]	-80 [-100, -27]
**31**	-38 [-48, -28]	-76 [-94, -19]	**0.59**	-8 [-26, 10]	-51 [-74, 5]
**16**	-38 [-53, -20]	-70 [-89, -12]	**1.12**	-11 [-27, 8]	-55 [-79, 7]
**8.16**	-27 [-38, -18]	-69 [-85, -18]	**2.17**	-13 [-32, 6]	-67 [-86, -20]
**4.19**	-21 [-34, -4]	-66 [-83, -9]	**4.19**	-21 [-38, -1]	-65 [-89, -13]
**2.17**	-13 [-26, -3]	-53 [-75, 7]	**8.16**	-26 [-47, -3]	-69 [-92, -20]
**1.12**	-9 [-22, 8]	-49 [-72, 12]	**16**	-37 [-54, -17]	-76 [-96, -29]
**0.59**	8 [-1, 20]	-9 [-51, 56]	**31**	-37 [-58, -16]	-70 [-94, -20]
**60.8(last)**	-37 [-51, -18]	-71 [-93, -13]	**60.8(last)**	-40 [-56, -20]	-76 [-97, -28]

Supplementary Table 7. Differences in 50IT between young subjects in the pinhole experiment and old subjects in the luminance experiment. The results are presented for all the pinhole conditions of each experimental session (s2b and b2s), and for luminance condition 1 (60.8 cd/m^2) of both sessions (b2d and d2b).

		**Young, no pinhole (first)**	**Young, 1 mm**	**Young, 2 mm**	**Young, 3 mm**	**Young, 4 mm**	**Young, 5 mm**	**Young, no pinhole (last)**
**b2s pinhole**	**(Old, b2d session)**	-43 [-56, -27]	32 [17, 45]	-14 [-39, 16]	-14 [-41, 11]	-21 [-45, -5]	-34 [-54,-15]	-22 [-42, -5]
	**(Old, d2b session)**	-47 [-64, -30]	28 [12, 47]	-18 [-44, 16]	-18 [-45, 9]	-25 [-50, -6]	-39 [-59,-14]	-26 [-48, -7]
**s2b pinhole**	**(Old, b2d session)**	-43 [-59, -26]	19 [-15, 36]	-27 [-41, -6]	-23 [-35, -6]	-28 [-48, -4]	-16 [-38, 5]	-36 [-53, -18]
	**(Old, d2b session)**	-47 [-65, -27]	14 [-16, 37]	-32 [-47, -7]	-27 [-40, -7]	-32 [-55, -6]	-20 [-43, 1]	-41 [-58, -19]

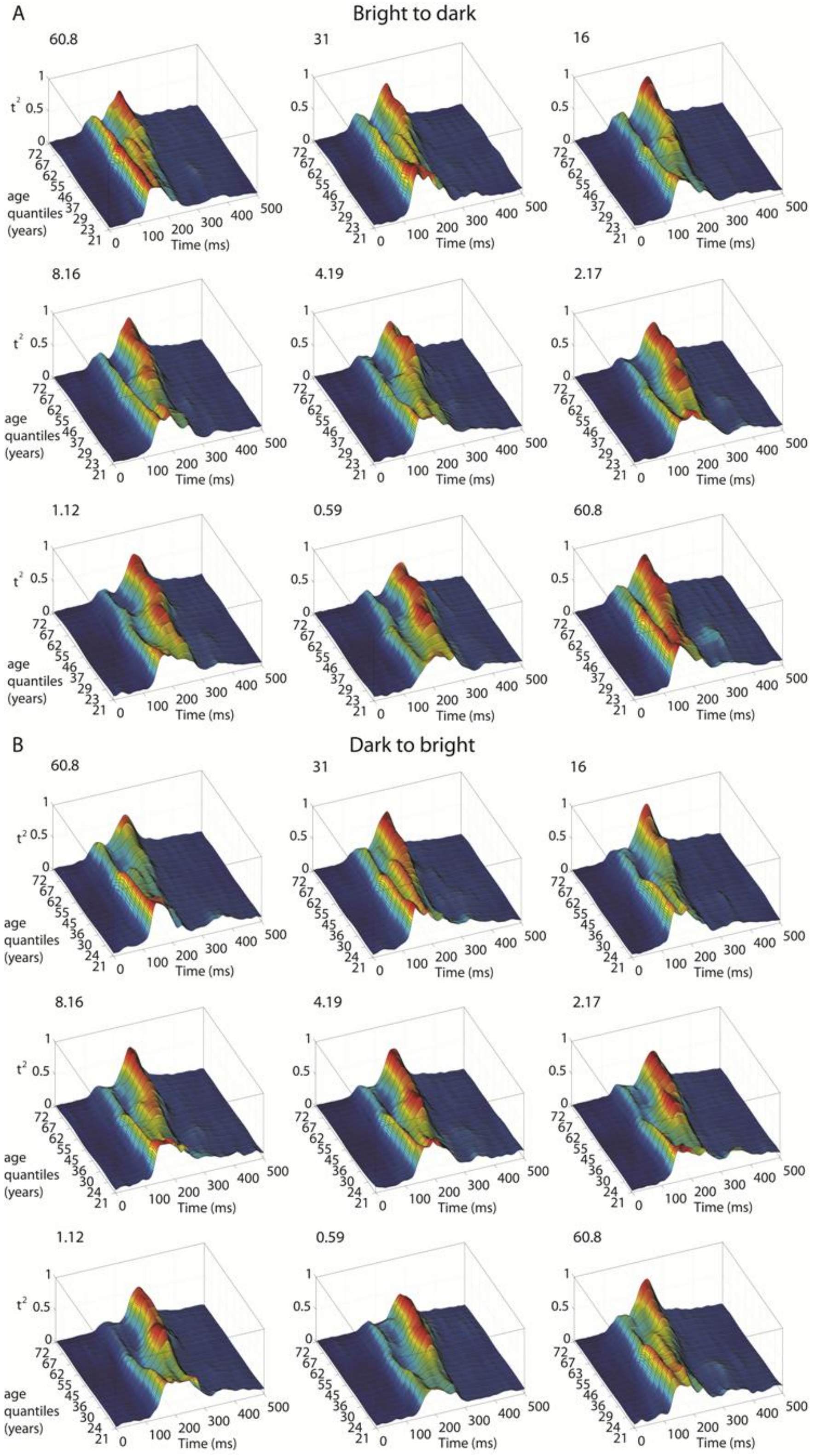

Supplementary Figure 1. 3D landscapes of t^2 functions. Each subplot depicts how the time-course (X axis) of normalized t^2 functions (Z axis) changes with age (Y axis), at the luminance indicated in the top left corner of the subplot. **(A)** B2d session. **(B)** D2b session. The process of generating the figure is described in section 2.6.1.2 of the article.

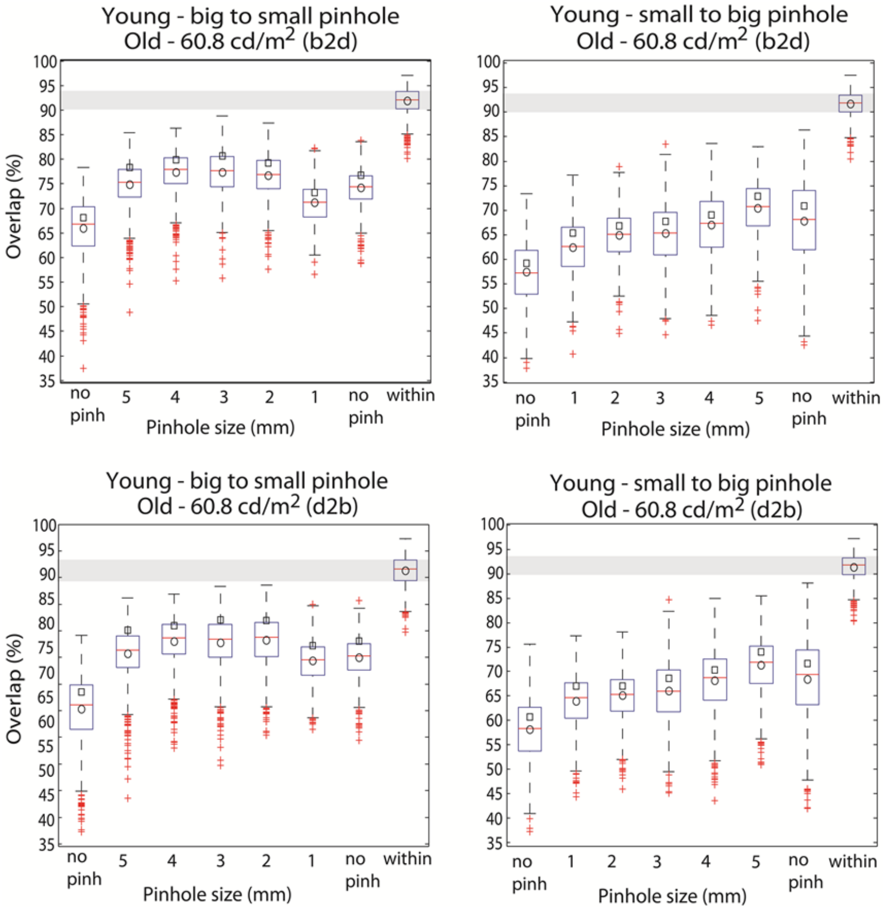

Supplementary Figure 2. Boxplots of t^2 function overlaps. Boxplots depicting distributions of t^2 function overlaps between young subjects in each pinhole condition and old subjects from the luminance experiment, in the brightest condition (60.8 cd/m^2) of b2d and d2b sessions. The last boxplot in each subplot shows the overlap within the group of old subjects.

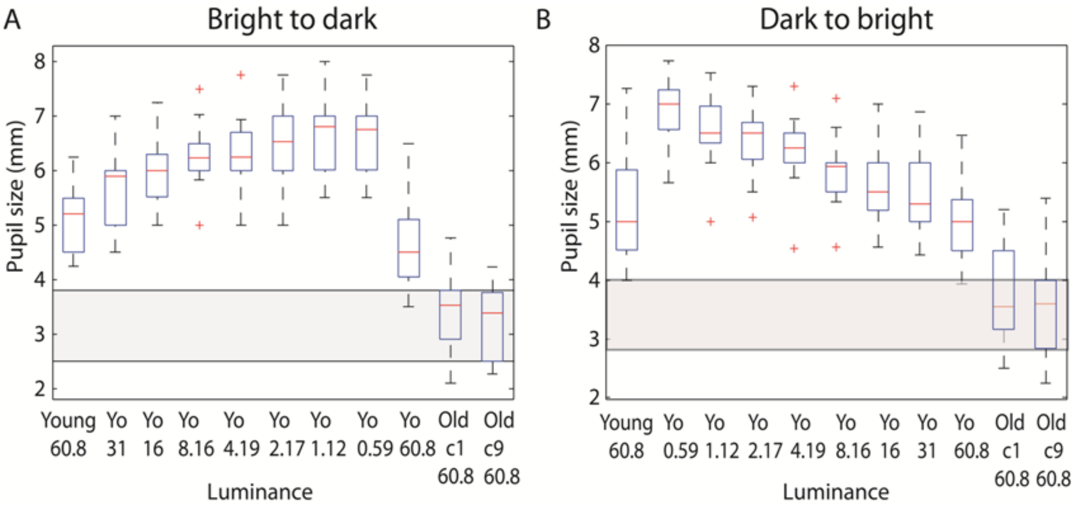

Supplementary Figure 3. Pupil size of young and old subjects. The first nine boxplots in each subplot depict the distributions of pupil sizes in young subjects, at nine luminances for b2d (A) and d2b (B) sessions. The last two boxplots in each subplot show results in old subjects in the two brightest conditions (luminance=60.8 cd/m^2).

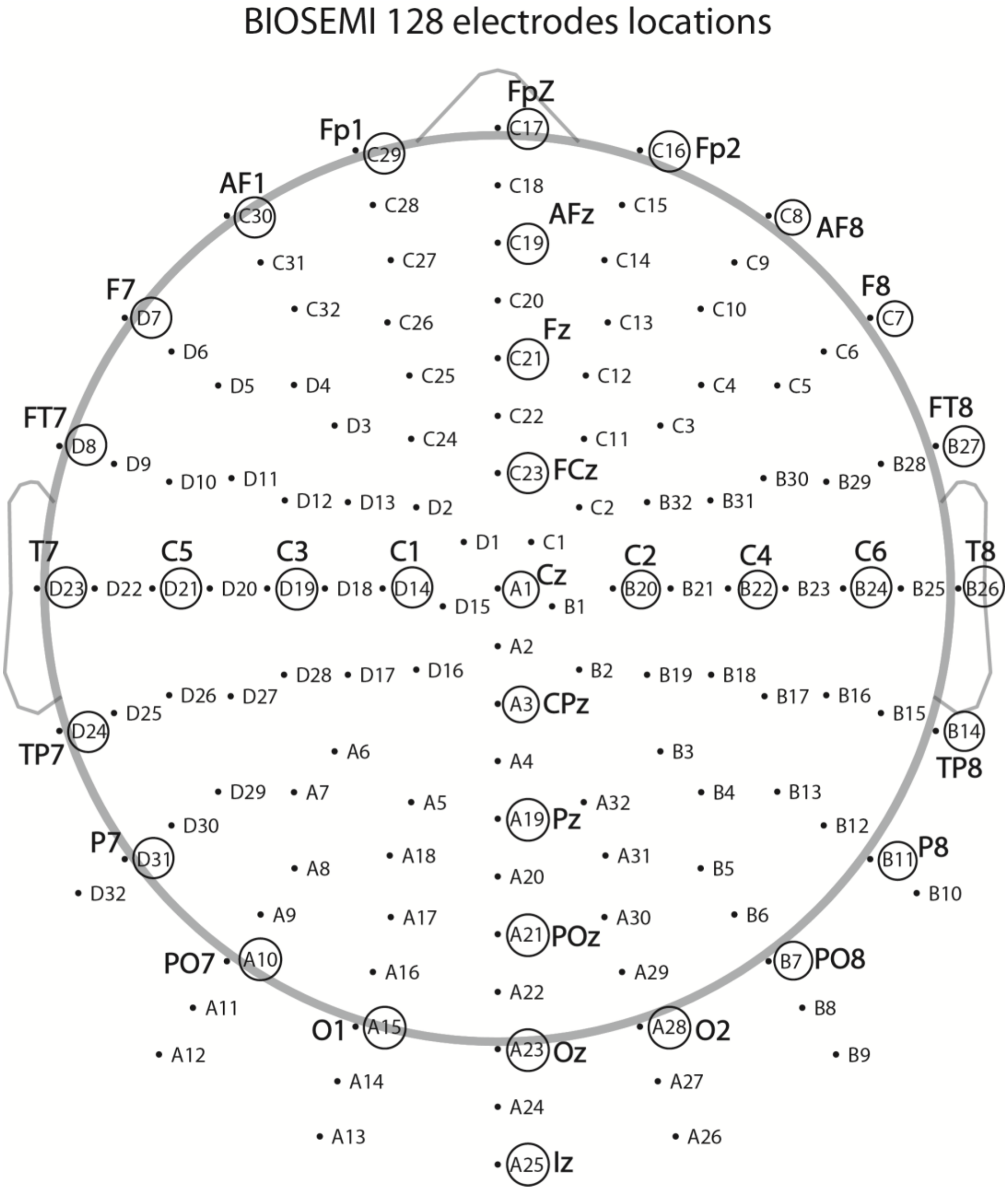

Supplementary Figure 4. Electrode montage with corresponding labelling from the 10/10 system (circled electrodes).
